# Integrative multi-omics analysis identifies a CMA-associated heterogeneity risk score and a cDCs-based immune score for robust prognostic stratification in colon cancer with single-center and experimental validation

**DOI:** 10.3389/fimmu.2026.1838405

**Published:** 2026-06-03

**Authors:** Jiaxing Zhang, Xiaodan Zhao, Yong Wang

**Affiliations:** 1 Department of General Surgery, The People’s Hospital of China Medical University, The People’s Hospital of Liaoning Province, Shenyang, China; 2 Department of General Surgery, The Fourth Affiliated Hospital of China Medical University, Shenyang, China

**Keywords:** chaperone-mediated autophagy, colon cancer, machine learning, prognostic signature, single-cell RNA sequencing, tumor immune microenvironment

## Abstract

**Background:**

Chaperone-mediated autophagy (CMA) plays an important role in tumor progression and remodeling of the tumor immune microenvironment. However, its functional heterogeneity, immune associations, and clinical significance in colon cancer remain unclear.

**Methods:**

Single-cell and bulk transcriptomic data were integrated to characterize CMA-related features in colon cancer. At the single-cell level, CMA activity was assessed across cell types, and differentially expressed genes between CMA-high and CMA-low groups were identified in myeloid subpopulations. Robust candidates were screened by recurrence frequency. At the bulk level, TCGA-COAD was used as the primary training cohort, and GSE17538 and GSE38832 for cross-cohort performance evaluation. CMA activity was quantified by ssGSEA, and candidates were further refined by WGCNA and tumor-normal differential expression analysis. An ensemble machine learning framework incorporating 101 algorithm combinations was used to construct a dual prognostic system consisting of the Risk Score and the Immune Risk Score. Prognostic performance was evaluated by Kaplan-Meier analysis, time-dependent ROC curves, and Cox regression. The Immune Risk Score was additionally evaluated in an independent single-center transcriptome cohort from Liaoning Central Hospital. MAPKAPK3 was identified as a key functional gene and validated *in vitro*. Drug sensitivity was predicted using pRRophetic and CGP2016.

**Results:**

CMA activity showed marked intercellular heterogeneity and was predominantly enriched in myeloid cells. Frequency-based screening, WGCNA, and tumor-normal differential expression analysis identified robust CMA-related candidates. The Risk Score showed favorable prognostic performance and generalizability across cohorts. The Risk Score remained an independent prognostic factor, whereas the Immune Risk Score functioned as an integrated prognostic score combining clinicopathologic and immune microenvironmental information. Immune analyses revealed consistent differences in regulatory T cells and resting dendritic cells across risk groups, suggesting an association between CMA-related risk states and an immunosuppressive microenvironment. The three-variable clinicomicroenvironmental model showed good predictive performance. MAPKAPK3 overexpression promoted proliferation, migration, and invasion of colon cancer cells, providing preliminary gain-of-function evidence for its tumor-promoting role.

**Conclusions:**

This study revealed CMA-related heterogeneity and immune microenvironmental features in colon cancer and established a robust dual prognostic system. MAPKAPK3 may serve as a key functional gene associated with tumor progression and microenvironment remodeling within the CMA-related prognostic framework.

## 1 Introduction

Colon cancer (COAD) is one of the leading malignant tumors of the digestive system worldwide in terms of both incidence and mortality (1). Despite continuous advances in comprehensive treatment strategies centered on surgery and combined with chemotherapy, targeted therapy, and immunotherapy, overall survival (OS) remains markedly heterogeneous among patients (2, 3). The traditional tumor-node-metastasis (TNM) staging system is primarily based on anatomical features for risk stratification. Although it can reflect disease progression to some extent, it still has clear limitations in predicting individualized prognosis and guiding precision treatment (4, 5). This limitation indicates that conventional clinicopathological parameters alone are often insufficient to fully capture the complex molecular heterogeneity of COAD and its microenvironment-dependent characteristics. Therefore, identifying more robust and biologically interpretable prognostic biomarkers from a multi-omics perspective is of substantial importance for improving risk stratification and therapeutic decision-making in COAD (6, 7).

The tumor immune microenvironment (TIME) plays a critical role in the initiation, progression, metastasis, and treatment response of COAD (8, 9). In recent years, chaperone-mediated autophagy (CMA), a highly selective pathway of protein degradation, has increasingly been recognized as an important regulator not only of intracellular protein homeostasis and metabolic reprogramming in tumor cells, but also of dynamic remodeling of the local immune microenvironment (10, 11). Previous studies have shown that aberrant activation of CMA can enhance the adaptability of tumor cells to stress conditions and may contribute to remodeling of the tumor microenvironment through its effects on inflammatory signaling, immune regulation, and cellular functional state (12). In particular, autophagy-related programs are closely linked to the functional remodeling of myeloid cells, such as macrophages, monocytes, and dendritic cells (13, 14). However, current studies on CMA in COAD remain largely limited to individual molecules or *in vitro* phenotypes (15). Systematic analyses of its heterogeneous distribution, cell-subpopulation associations, and immune infiltration patterns at single-cell resolution are still lacking. As a result, the biological significance and potential clinical value of CMA in COAD have not yet been fully elucidated.

On the other hand, prognostic modeling has become an important direction in precision medicine for COAD (16, 17). However, most existing models still rely mainly on a single statistical method or a single machine learning algorithm, such as least absolute shrinkage and selection operator (LASSO) or Cox regression, and are often developed using limited sample sizes or single-source cohorts (18). These models are therefore susceptible to data structure bias, platform differences, and cohort-specific effects, which may lead to overfitting, limited generalizability, and reduced performance in cross-cohort evaluation (19, 20). For a biologically heterogeneous disease such as COAD, reliance on a single algorithm is unlikely to adequately capture its complex risk landscape (21). Accordingly, large-scale ensemble machine learning frameworks, which systematically compare multiple algorithms and their combinations, have emerged as an effective strategy for improving model robustness and translational potential (22). Nevertheless, to the best of our knowledge, no previous study has systematically integrated single-cell characterization of CMA-related heterogeneity with large-scale ensemble machine learning, cross-cohort evaluation, and experimental verification in COAD.

In this context, we performed an integrative analysis combining single-cell transcriptomic data with large-scale bulk transcriptomic cohorts to systematically investigate CMA-related heterogeneity in COAD. First, we characterized the distribution of CMA activity across different cell types, particularly within myeloid cell subpopulations, at single-cell resolution, and identified robust CMA-related candidate genes through differential expression analysis. We then integrated weighted gene co-expression network analysis (WGCNA) and tumor-normal differential expression analysis to refine key features associated with CMA activity at the bulk level. Based on these candidates, we introduced an ensemble machine learning framework incorporating 101 algorithm combinations and established a dual prognostic evaluation system consisting of the Risk Score and the Immune Risk Score. The Risk Score was evaluated across TCGA-COAD and GEO cohorts, whereas the cDC-related immune component of the integrated score was further assessed in a single-center transcriptomic cohort from Liaoning Central Hospital. In addition, we further characterized the tumor immune microenvironment under different risk states and developed a more clinically applicable clinical-microenvironment integrated model, which was externally validated in an independent cohort from Liaoning Central Hospital. Finally, by integrating dual-score correlation analyses with immune infiltration analyses, we identified MAPKAPK3 as a key functional gene. Its biological role was further validated through *in vitro* experiments. Overall, this study aimed to clarify the biological significance of CMA in COAD from the perspectives of single-cell heterogeneity, robust modeling, and experimental validation, and to provide a new basis for risk stratification and individualized therapeutic exploration.

## 2 Materials and methods

### 2.1 Data acquisition and preprocessing

This study integrated publicly available single-cell transcriptomic data, bulk transcriptomic data, and corresponding clinical follow-up information. The scRNA-seq dataset was obtained from the human colon cancer single-cell atlas available from the Gene Expression Omnibus (GEO) database under accession number GSE178341 and the Broad Single Cell Portal. The corresponding expression matrix and cell annotation information were used for downstream single-cell analysis. At the bulk transcriptomic level, the The Cancer Genome Atlas (TCGA) COAD cohort was used as the training set, whereas the Gene Expression Omnibus (GEO) cohorts GSE17538 and GSE38832 were used as external validation sets. All included samples had available expression matrices and OS information.

After harmonizing the expression matrices across different cohorts, shared genes were extracted, and batch effects were corrected using the empirical Bayes-based ComBat method to reduce systematic bias arising from different platforms and data sources (23).

### 2.2 Construction of the CMA gene set and scRNA-seq analysis

To quantify CMA-related activity at the transcriptomic level, we curated a gene set associated with the positive regulation, negative regulation, and effector functions of CMA-associated autophagy, based on previous reports and recent single-cell evidence showing tissue- and cell-type-specific heterogeneity of CMA activity (24). For each cell or bulk sample, genes in the three functional categories were extracted and summarized, and the CMA-related transcriptomic score was calculated using the following weighted formula: CMA_score = (2 × effector gene expression sum − negative regulator gene expression sum + positive regulator gene expression sum)/total number of genes included in the signature. This score was used for single-cell CMA-related activity evaluation, bulk-level ssGSEA analysis, and WGCNA module-trait correlation analysis. Because the score was derived from transcriptomic expression profiles, it was interpreted as a CMA-related transcriptomic proxy rather than a direct measurement of CMA flux.

The scRNA-seq data were mainly analyzed using the Seurat package (25). After normalization, dimensionality reduction, and clustering, major cell populations were annotated based on reference annotation files and canonical marker genes, including epithelial cells, myeloid cells, T/NK/ILC cells, B cells, plasma cells, stromal cells, and mast cells. To reduce batch effects among different samples, data integration was performed using the Harmony algorithm, and visualization was conducted by t-distributed stochastic neighbor embedding (t-SNE) or uniform manifold approximation and projection (UMAP).

Because CMA activity appeared to be more pronounced in myeloid cells, myeloid cells were further re-clustered to identify their internal subgroup structure and to compare CMA states among different subpopulations.

### 2.3 Identification of CMA-related DEGs at the single-cell level

Within each myeloid cell subpopulation, cells were divided into CMA-high and CMA-low groups according to their CMA activity scores, and differentially expressed genes (DEGs) between the two groups were identified. This analysis was performed independently within each subpopulation. The frequency of occurrence of each DEG across all myeloid subpopulations was then calculated. Based on the frequency distribution, high-frequency genes were displayed. To reduce the influence of single-subpopulation-specific noise and prioritize recurrent CMA-related transcriptional features for downstream bulk-level modeling, genes appearing in at least two myeloid subpopulations were defined as robust CMA-related candidate genes.

### 2.4 WGCNA and candidate gene integration

WGCNA was performed using the TCGA-COAD cohort (26). First, an appropriate soft-thresholding power was selected according to the scale-free topology criterion to construct a weighted co-expression network. Distinct co-expression modules were then identified using the topological overlap matrix and the dynamic tree cut algorithm. Module eigengenes were subsequently correlated with the bulk-level CMA-related transcriptomic score to identify co-expression modules associated with CMA-related transcriptomic variation. Based on module membership, gene significance, and overall result stability, the turquoise module was selected as the key module.

Differential expression analysis between tumor and normal tissues was further performed. Tumor samples were obtained from 420 tumor tissues in the TCGA-COAD cohort, whereas normal samples consisted of adjacent normal tissues from TCGA and normal colon tissues from the Genotype-Tissue Expression (GTEx) database, yielding a total of 820 normal samples. The genes in the key turquoise module were intersected with tumor-normal DEGs to generate the candidate gene set used for subsequent risk model construction.

### 2.5 Construction of a CMA-related prognostic model using ensemble machine learning

Based on the candidate genes identified above, an ensemble machine learning framework was introduced to systematically compare multiple classical survival analysis algorithms and their combinations. The included algorithms and modeling strategies comprised random survival forest (RSF), elastic net (Enet), stepwise Cox regression (StepCox), CoxBoost, partial least squares Cox regression (plsRcox), SuperPC, gradient boosting machine (GBM), survival support vector machine (survival-SVM), ridge regression, and least absolute shrinkage and selection operator (Lasso), resulting in a total of 101 modeling combinations.

The TCGA-COAD cohort was used as the primary training cohort, whereas GSE17538 and GSE38832 were used as cross-cohort evaluation cohorts. Harrell’s concordance index (C-index) was used to evaluate the predictive performance of different algorithm combinations, and the optimal model was selected based on the average cross-cohort C-index. A Risk Score was then constructed using the final model, and patients were stratified into high-risk and low-risk groups according to the median score.

### 2.6 Survival analysis and evaluation of independent prognostic value

Kaplan-Meier analysis was used to compare OS between different risk groups, and statistical significance was assessed using the log-rank test. Predictive performance at different time points was quantified using time-dependent receiver operating characteristic (ROC) curves (27).

The Risk Score, Immune Risk Score, and conventional clinicopathological parameters were jointly included in univariate and multivariate Cox proportional hazards regression analyses to evaluate the independent prognostic value of different scoring systems. For some clinical variables, category consolidation was performed according to clinical relevance before inclusion in the Cox models.

### 2.7 Tumor immune microenvironment analysis

The quanTIseq algorithm implemented in the immunedeconv package was used to estimate the infiltration abundance of different immune cell types in the TCGA cohort (28). Myeloid-related components, including macrophage M1, macrophage M2, monocyte, neutrophil, and myeloid dendritic cell populations, were selected and summed to calculate the CMA-Myeloid score.

In addition, CIBERSORT and IOBR were applied to quantify the relative abundance of immune cell subsets in the tumor microenvironment and to compare infiltration differences between different risk groups. Meanwhile, immune-related pathways were scored using ssGSEA to evaluate differences in immune functional states across distinct risk conditions (29).

### 2.8 Construction and external validation of the immune risk score

Given that high-dimensional risk models based on bulk and single-cell transcriptomic data may show favorable predictive performance but remain limited in current clinical practice because of high testing costs and restricted accessibility, an Immune Risk Score reflecting the tumor immune microenvironment was further developed.

First, bulk transcriptomic data were deconvoluted using the xCell algorithm to estimate the infiltration abundance of different immune and stromal cell subsets (30). These microenvironmental features, together with conventional clinical parameters, including TNM stage, age, and sex, were incorporated into LASSO Cox regression analysis in the TCGA cohort to identify the most parsimonious prognostic variable combination. The Immune Risk Score was then established based on the selected clinicopathological and immune microenvironmental variables and was interpreted as a clinicopathological-immune integrated prognostic score. According to the median value of the Immune Risk Score, patients were divided into high-risk and low-risk groups.

After model establishment, Kaplan-Meier curves and time-dependent ROC curves were used in the TCGA cohort to evaluate its prognostic stratification ability and predictive performance. The model was then applied to an independent single-center transcriptome cohort from Liaoning Central Hospital. In this cohort, cDC abundance was inferred from transcriptomic profiles using the xCell algorithm rather than measured directly by flow cytometry or immunohistochemistry.

### 2.9 Comparison of the dual scoring systems, pathway analysis, and key gene selection

Correlation analysis was performed to assess the relationship between the Risk Score and the Immune Risk Score, and univariate as well as multivariate Cox regression analyses were conducted to compare the prognostic value of these two scoring systems.

Shared and distinct pathways associated with the two scoring systems were compared based on gene set variation analysis (GSVA) or Hallmark gene sets, followed by joint comparisons between the double-high and double-low subgroups. Correlations between signature genes and the two risk scores were further calculated. Candidate genes were then comprehensively evaluated in combination with immune cell infiltration correlations. Genes showing significant associations with both scoring systems and ranking highly among candidate features were selected as key targets for further validation.

### 2.10 *In vitro* functional validation of MAPKAPK3

Based on the preceding multi-omics integration analysis and machine learning screening results, MAPKAPK3, a key gene showing significant associations with the dual-risk model and close links to the immune microenvironment, was selected for *in vitro* functional validation.

The human colorectal cancer cell lines HCT116 and LoVo were cultured in medium containing 10% fetal bovine serum (FBS) at 37 °C in a humidified incubator with 5% carbon dioxide (CO_2_). Flag-tagged MAPKAPK3 overexpression plasmids were transfected into cells using Lipofectamine 3000 (Invitrogen) to construct the overexpression model (OE-MAPKAPK3), while empty vector-transfected cells served as the control group (OE-NC). Cells were collected 48 h after transfection for subsequent experiments. Western blotting was used to assess MAPKAPK3 protein expression and verify transfection efficiency. Cell proliferation was evaluated using the CCK-8 assay. Cell migration was examined by wound-healing assay. Cell migratory and invasive abilities were assessed by Transwell assays, with Matrigel pre-coating for invasion assays. Colony formation assays were performed to evaluate long-term proliferative capacity. All *in vitro* experiments were conducted at least in triplicate.

### 2.11 Drug sensitivity prediction

The R package pRRophetic and the Cancer Genome Project 2016 (CGP2016) drug sensitivity database, namely Genomics of Drug Sensitivity in Cancer, were used to predict the sensitivity of samples in all cohorts to multiple anticancer drugs (31). Drug sensitivity was quantified by the estimated half-maximal inhibitory concentration (IC50) (32). Based on the Wilcoxon rank-sum test, drug sensitivity differences between the high-risk and low-risk groups defined by the Risk Score and the Immune Risk Score were compared separately. Candidate drugs showing consistent differences in both scoring systems were further screened to identify subgroups that might derive differential therapeutic benefit.

### 2.12 Statistical analysis

All statistical analyses and graphical visualizations were performed in the R environment. For continuous variables, Student’s t-test was used for comparisons between groups when data were normally distributed, whereas the Wilcoxon rank-sum test was applied for non-normally distributed data. Differences among multiple groups were analyzed using the Kruskal-Wallis test. Correlations between variables were assessed using Spearman’s rank correlation coefficient. Unless otherwise specified, all statistical tests were two-sided, and a *P* value < 0.05 was considered statistically significant (*P* < 0.05, *P* < 0.01, *P* < 0.001).

## 3 Results

**Figure 1:**
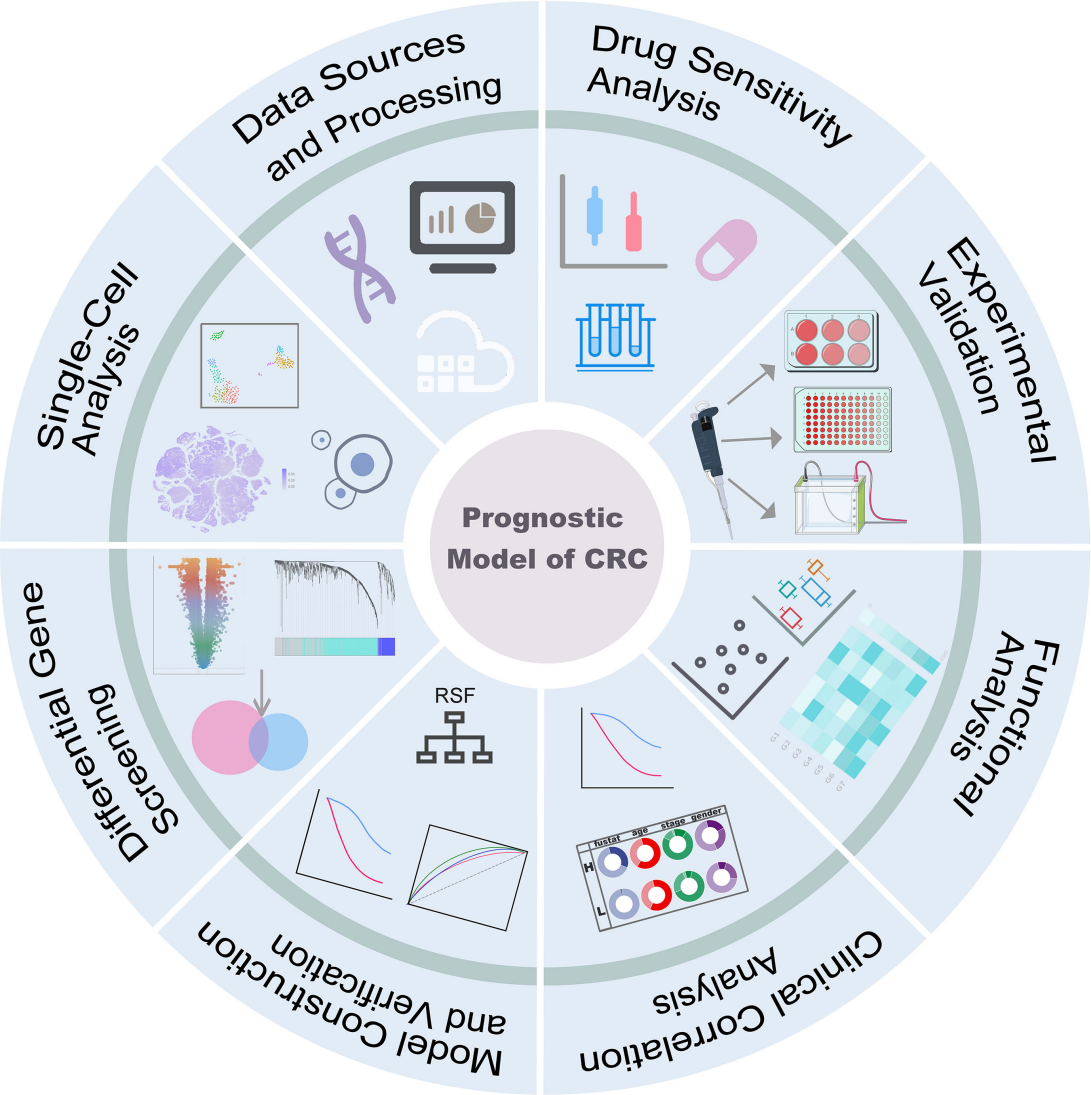
Study flowchart.

All analytical processes are illustrated in the flowchart (**Figure 1**).

### 3.1 Single-cell landscape of the COAD tumor microenvironment and cellular heterogeneity of CMA-related features

**Figure 2:**
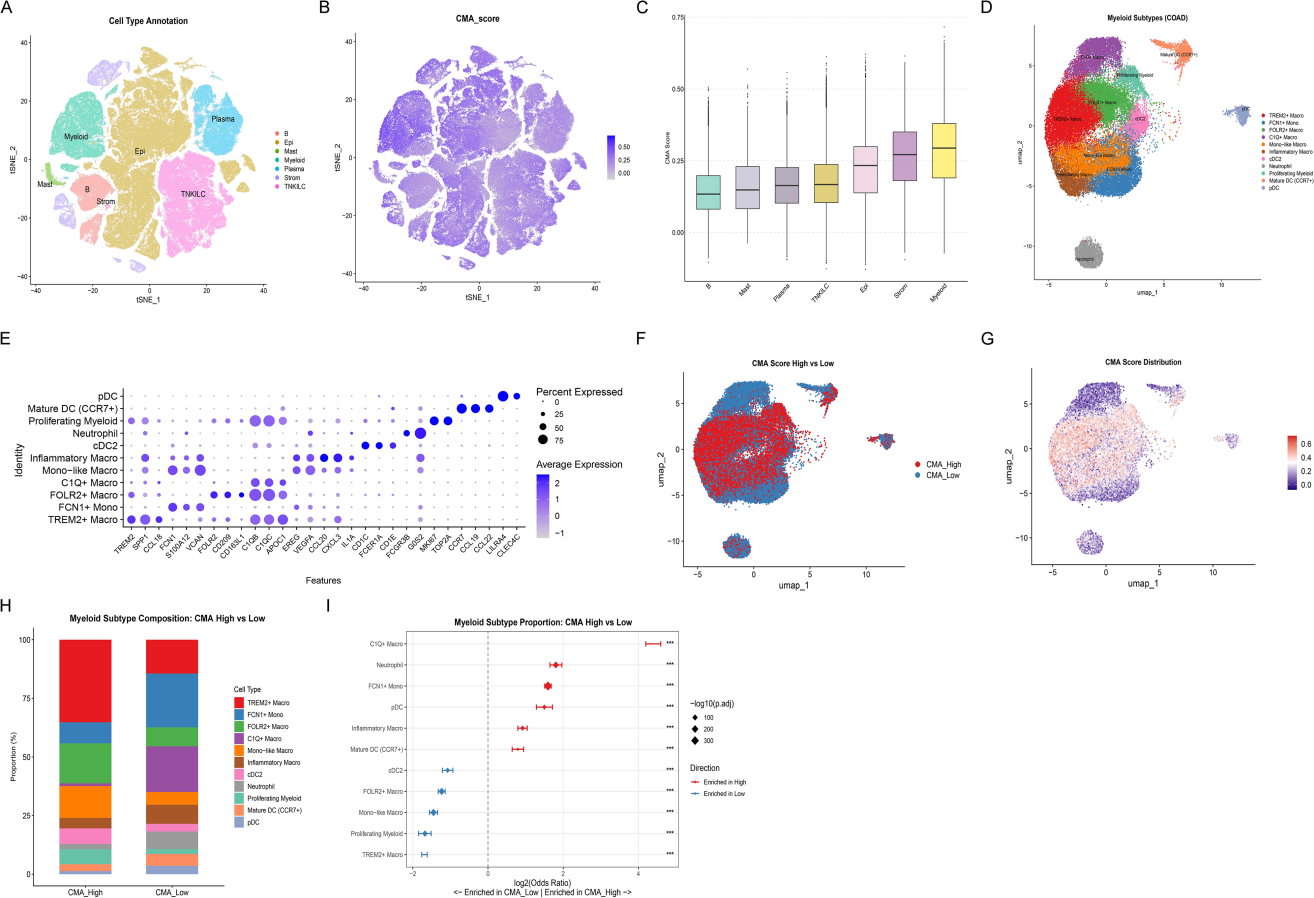
Single-cell landscape of the COAD tumor microenvironment and distribution of CMA-related features. **(A)** t-SNE plot showing the major cell populations in COAD samples. **(B)** t-SNE plot showing the distribution of CMA scores across all cells. **(C)** Comparison of CMA scores among major cell types. **(D)** UMAP plot showing re-clustering of myeloid cells into distinct subpopulations. **(E)** Bubble plot showing the expression of marker genes across myeloid subpopulations. **(F)** UMAP plot of myeloid cells stratified into CMA-High and CMA-Low groups. **(G)** UMAP plot showing the distribution of CMA scores within myeloid cells. **(H)** Relative proportions of myeloid subpopulations in the CMA-High and CMA-Low groups. **(I)** Forest plot showing the enrichment preference of myeloid subpopulations between the CMA-High and CMA-Low groups. (***P < 0.001).

At single-cell resolution, dimensionality reduction and clustering analysis of COAD (COAD) tumor samples revealed a well-defined topological distribution of cells in low-dimensional space. Based on canonical marker gene expression, the cells were annotated into seven major populations, including Epi, Myeloid, TNKILC, B, Plasma, Strom, and Mast. These populations were relatively well separated in the t-SNE space, with clear inter-cluster boundaries, thereby outlining the cellular landscape of the COAD tumor microenvironment (**Figure 2A**).

On this basis, a CMA activity score was calculated at the single-cell level by quantifying the expression of a predefined gene set. Projection of the CMA score onto the t-SNE space showed marked spatial heterogeneity. High-score regions were not uniformly distributed across all cell populations but instead exhibited clear lineage preference (**Figure 2B**). Quantitative comparison of CMA scores across cell types further demonstrated that myeloid cells displayed the highest CMA score, with both the median value and overall distribution markedly higher than those of stromal cells, epithelial cells, and TNKILC cells. In contrast, B cells and mast cells maintained relatively low CMA scores (**Figure 2C**). These results suggest that CMA-related biological features are predominantly enriched in the myeloid compartment of the COAD microenvironment.

Given the prominent enrichment of CMA-related features in myeloid cells, we further performed refined re-clustering analysis of this compartment. Based on transcriptomic differences, myeloid cells were subdivided into 11 subpopulations, including immunoregulatory TREM2+ Macro, C1Q+ Macro, and FOLR2+ Macro, as well as inflammation-associated FCN1+ Mono and Inflammatory Macro, among others (**Figure 2D**). The bubble plot showed highly specific marker gene expression patterns across these subpopulations. For example, TREM2+ Macro highly expressed TREM2 and SPP1, whereas C1Q+ Macro specifically expressed the complement-related genes C1QB and C1QC, supporting the accuracy of the subgroup annotation (**Figure 2E**).

Further projection of the CMA score onto the UMAP space of myeloid cells showed that CMA states remained highly heterogeneous even within the same lineage. The distribution of CMA scores was not uniform across myeloid subpopulations, but instead displayed gradient-like patterns centered on specific subgroups (**Figures 2F, G**). To further characterize the source of this heterogeneity, myeloid cells were stratified into CMA-High and CMA-Low groups, followed by systematic analysis of subgroup composition (**Figure 2H**) and odds ratios (**Figure 2I**). Cell composition analysis showed marked remodeling of myeloid subpopulations between the two CMA states. In the CMA-High group, TREM2+ Macro remained the dominant population, while FOLR2+ Macro, which is characterized by tissue-resident features, showed an evident enrichment trend. Forest plot analysis based on odds ratios further statistically confirmed this observation, showing that FOLR2+ Macro was significantly enriched in the CMA-High group (odds ratio > 1, *P* < 0.001). In contrast, the CMA-Low group exhibited a distinct inflammatory profile, characterized by a substantial increase in the proportion of FCN1+ Mono and a higher abundance of complement-activated C1Q+ Macro. The forest plot results consistently supported this pattern, with both FCN1+ Mono and C1Q+ Macro showing odds ratios significantly biased toward the CMA-Low group. Notably, FOLR2+ Macro and C1Q+ Macro displayed opposite enrichment trends in the forest plot, suggesting a mutually exclusive distribution pattern between these two subpopulations. Together, these findings indicate that different CMA activity states are associated with distinct remodeling patterns of myeloid cell composition in COAD.

### 3.2 Identification of core CMA-related feature genes shared across lineages

**Figure 3:**
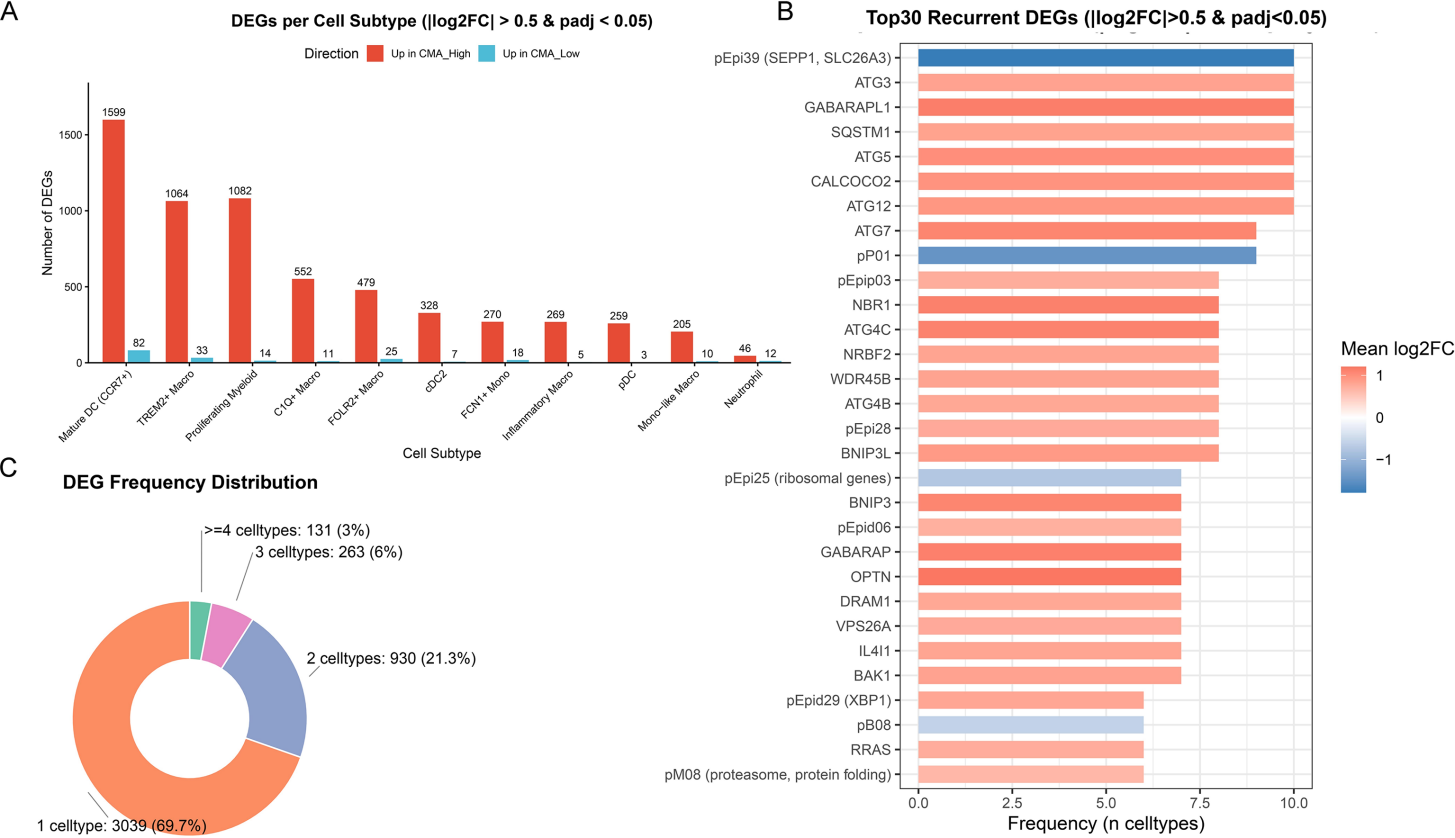
Identification and distribution of recurrent DEGs between CMA-High and CMA-Low myeloid populations. **(A)** Number of up-regulated (red) and down-regulated (blue) DEGs identified in each myeloid subtype. **(B)** Bar plot showing the top 30 recurrent DEGs ranked by their occurrence across different cell subtypes. Color scale represents the mean log2 fold change. **(C)** Donut chart showing the frequency distribution of DEGs across different cell subtypes, highlighting the cell-type specificity of CMA-related transcriptional changes.

To further investigate the molecular alterations driven by CMA-related features at the transcriptomic level, we performed differential expression analysis between the CMA-High and CMA-Low groups across 11 myeloid subpopulations. The results showed marked heterogeneity in the transcriptional response to changes in CMA states across different cell subpopulations. Among them, Mature DC (CCR7+), TREM2+ Macro, and Proliferating Myeloid exhibited the most pronounced transcriptomic remodeling, with substantially more differentially expressed genes than other cell types, and most of these genes were upregulated in the CMA-High group (**Figure 3A**).

Subsequently, to identify key molecular features shared across cell lineages, we calculated the recurrence frequency of differentially expressed genes across subpopulations and focused on the top 30 most frequently occurring genes. The analysis showed that, except for a few downregulated genes, such as pEpi39, most high-frequency differentially expressed genes displayed significant upregulation in the CMA-High group. Notably, core autophagy-related genes, including ATG3, GABARAPL1, SQSTM1, ATG5, CALCOCO2, and ATG12, ranked among the top candidates and were commonly upregulated across more than 10 myeloid subpopulations (**Figure 3B**). The enrichment of these core molecules supported the relevance of the CMA-related transcriptomic scoring strategy and suggested enhanced autophagy- and lysosome-associated transcriptional programs in the myeloid microenvironment under high-CMA-related conditions. Further frequency distribution analysis showed that although 69.7% of the differentially expressed genes were specifically expressed in only one subpopulation, a considerable proportion of genes were still shared across multiple subpopulations. To ensure the robustness of subsequent prognostic model construction and reduce interference from single-cell-specific noise, genes with a recurrence frequency greater than or equal to 2 were ultimately defined as the core CMA-related candidate gene set for subsequent prognostic feature screening (**Figure 3C**).

### 3.3 Screening of core CMA-related prognostic biomarkers based on WGCNA and tumor-normal differential expression analysis

**Figure 4:**
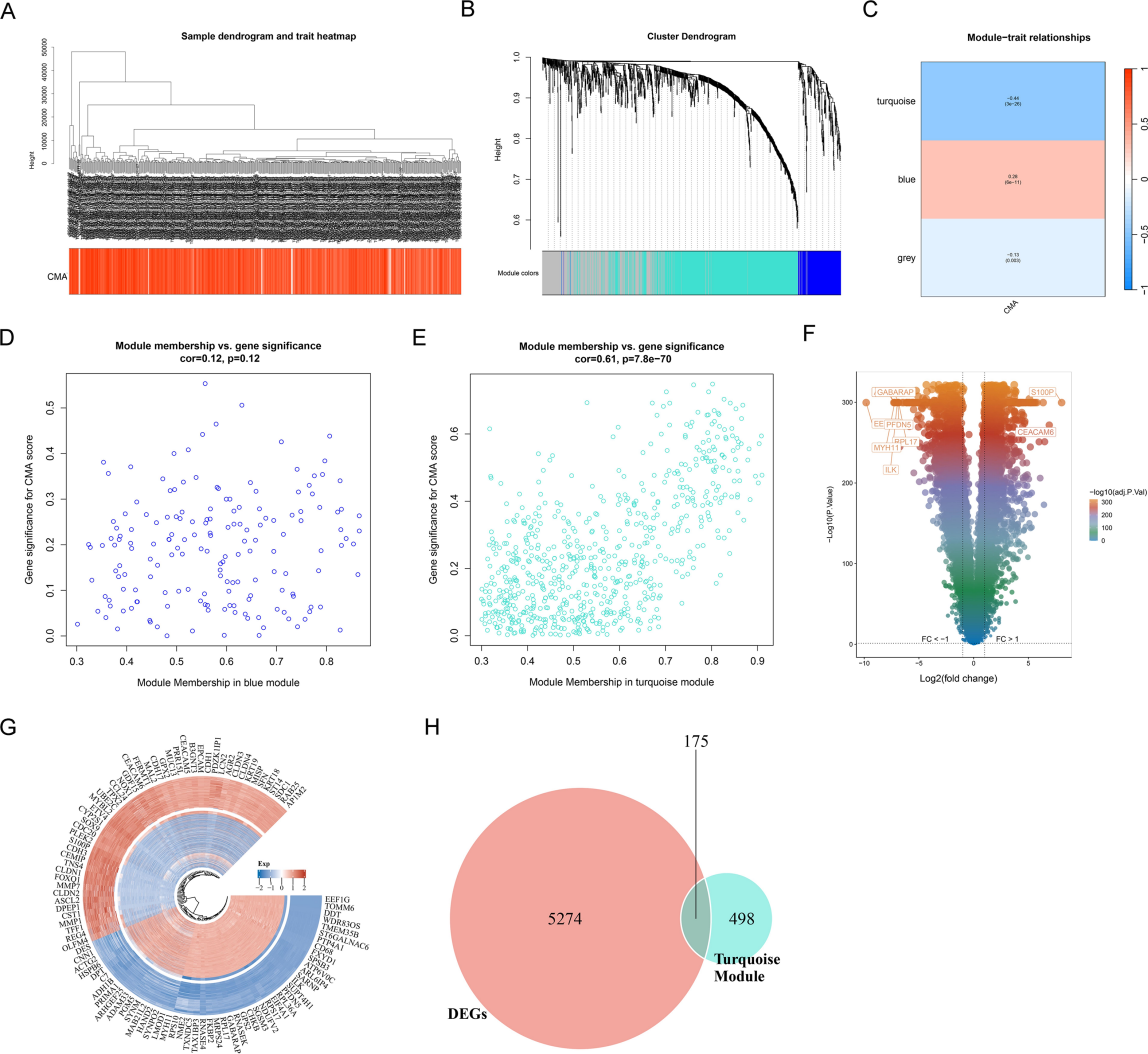
Identification of core CMA-related candidate genes by WGCNA and tumor-normal differential expression analysis. **(A)** Sample clustering dendrogram for WGCNA. **(B)** Gene dendrogram showing module assignment by WGCNA. **(C)** Heatmap of correlations between module eigengenes and CMA score. **(D)** Scatter plot of module membership versus gene significance in the blue module. **(E)** Scatter plot of module membership versus gene significance in the turquoise module. **(F)** Volcano plot of DEGs between tumor and normal samples. **(G)** Heatmap of representative DEGs between tumor and normal samples. **(H)** Venn diagram showing the overlap between turquoise module genes and tumor-normal DEGs.

To translate the CMA-related features identified at the single-cell level into clinically applicable prognostic biomarkers and to validate their robustness in a large cohort, we performed weighted gene co-expression network analysis (WGCNA) using transcriptomic data from the TCGA-COAD cohort. By constructing a scale-free co-expression network and hierarchical clustering, genes were classified into different co-expression modules (**Figures 4A, B**). The module-trait relationship heatmap showed that, among all identified modules, the turquoise module exhibited the most significant correlation with the CMA score (R = -0.44, *P* = 3e-26), indicating that the gene expression pattern within this module was highly coordinated with CMA biological activity (**Figure 4C**). Further analysis of the correlation between module membership and gene significance confirmed that genes in the blue module did not show significant correlation (cor = 0.12, *P* = 0.12) (**Figure 4D**), whereas the turquoise module displayed a high degree of functional consistency (cor = 0.61, *P* = 7.8e-70) (**Figure 4E**). Therefore, the 498 genes within the turquoise module were identified as key CMA-related co-expression genes.

Meanwhile, to ensure that the selected genes were biologically relevant in the development and progression of COAD, we integrated the TCGA-COAD cohort with the GTEx database and performed genome-wide differential expression analysis using a total of 420 tumor samples and 820 normal samples. The analysis revealed substantial transcriptomic remodeling in tumor tissues compared with normal tissues, with a total of 5,274 significantly differentially expressed genes identified. The distribution pattern of these genes was visualized in the volcano plot (**Figure 4F**). Among them, oncogenic genes such as S100P and CEACAM6 were significantly upregulated in tumor tissues (FC > 1), whereas key functional genes including GABARAP, PFDN5, ILK, and MYH11 were significantly downregulated (FC < -1). These genes showed markedly distinct expression abundance patterns between tumor and normal tissues, and their cancer-specific expression profiles were clearly illustrated in the heatmap (**Figure 4G**). To obtain robust candidate genes that were both closely associated with CMA activity and aberrantly expressed in tumor tissues, we intersected the genes identified in the turquoise module with the tumor-normal differentially expressed genes. The Venn diagram showed that 175 genes satisfied both criteria (**Figure 4H**). These 175 intersecting genes integrated both network topology features and disease specificity and were used as the candidate gene pool for subsequent construction of the CMA-related prognostic risk model in COAD.

### 3.4 Construction and validation of a robust CMA-related prognostic model based on an ensemble machine learning framework

**Figure 5:**
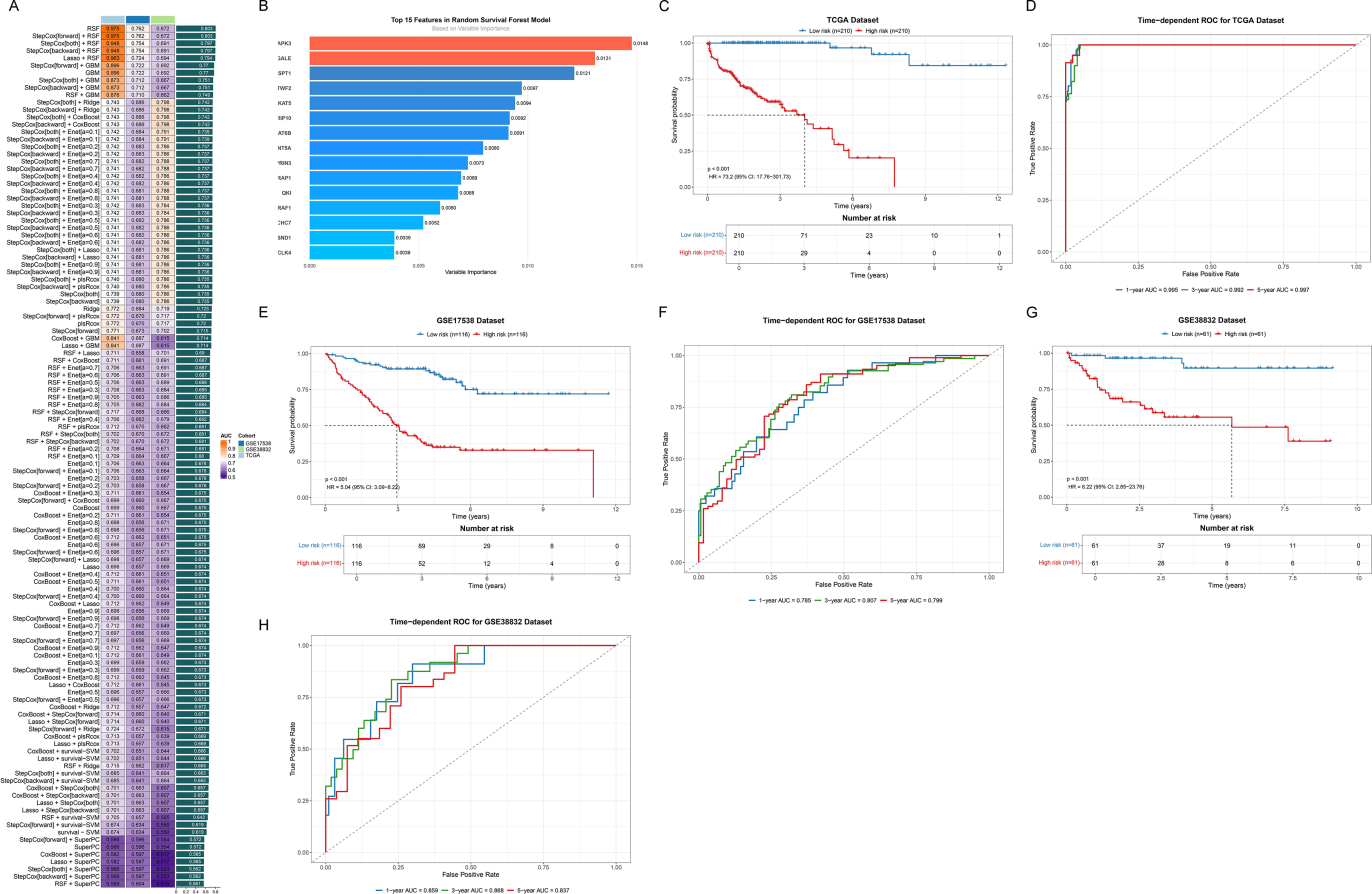
Construction and validation of the CMA-related prognostic model based on an integrative machine learning framework. **(A)** Evaluation of 101 machine learning algorithm combinations based on average cross-cohort C-index values across TCGA-COAD, GSE17538, and GSE38832, identifying RSF as the optimal predictive model. **(B)** Feature importance ranking (VIMP) of the candidate genes generated by the RSF algorithm, highlighting MAPKAPK3 and GALE as the top-ranked prognostic features. **(C)** Kaplan-Meier survival analysis of OS for patients in the high- and low-risk groups within the TCGA-COAD training cohort. **(D)** Time-dependent ROC curves demonstrating the predictive accuracy of the risk model for 1-, 3-, and 5-year OS in the TCGA-COAD cohort. **(E)** Kaplan-Meier survival curve of OS for patients in the GSE17538 evaluation cohort. **(F)** Time-dependent ROC curves for 1-, 3-, and 5-year OS in the GSE17538 cohort. **(G)** Kaplan-Meier survival curve of OS for patients in the GSE38832 evaluation cohort. **(H)** Time-dependent ROC curves for 1-, 3-, and 5-year OS in the GSE38832 evaluation cohort.

To further identify the most prognostically informative features from the 175 CMA-related candidate genes and to construct a robust clinical predictive model, we adopted an integrated screening strategy incorporating multiple machine learning algorithms. By performing full-process simulation and C-index evaluation across 101 different algorithm combinations generated from 10 machine learning algorithms, we found that RSF achieved the highest average cross-cohort C-index across TCGA-COAD, GSE17538, and GSE38832. This result indicated that RSF had the best performance in capturing the complex nonlinear relationship between CMA-related features and patient survival outcomes (**Figure 5A**). We then ranked the candidate genes according to variable importance (VIMP) using the RSF algorithm to identify the key genes contributing most to prognosis. The results showed that MAPKAPK3 had the highest VIMP score among all candidate features and was identified as the most critical prognostic predictor (**Figure 5B**). Based on these selected core feature genes, we established a CMA-related Risk Score model.

To comprehensively evaluate the clinical predictive performance of this scoring system, systematic validation was performed in multiple independent cohorts. First, in the TCGA training cohort, Kaplan–Meier survival analysis showed that patients in the high-risk group had significantly shorter OS than those in the low-risk group (*P* < 0.001) (**Figure 5C**). Time-dependent ROC curve analysis demonstrated that the model showed favorable accuracy in predicting 1-year, 3-year, and 5-year survival, with consistently high area under the curve (AUC) values (**Figure 5D**).

To further assess the cross-cohort robustness of the model, the scoring system was applied to two GEO evaluation cohorts, GSE17538 and GSE38832. In the GSE17538 cohort, survival analysis again confirmed a significant association between a high-Risk group and poor prognosis (*P* < 0.001) (**Figure 5E**), and the ROC curves showed that the model maintained stable predictive performance (**Figure 5F**). Similarly, in the GSE38832 cohort, the model was still able to effectively distinguish high-risk from low-risk patients (*P* < 0.001) (**Figure 5G**) and demonstrated strong predictive performance for survival at different time points (**Figure 5H**). Taken together, the CMA-related prognostic model identified by machine learning demonstrated strong robustness across multiple cohorts and validation settings, with promising potential for clinical application.

### 3.5 Association of the initial CMA risk model with clinical characteristics and stratified prognostic evaluation

**Figure 6:**
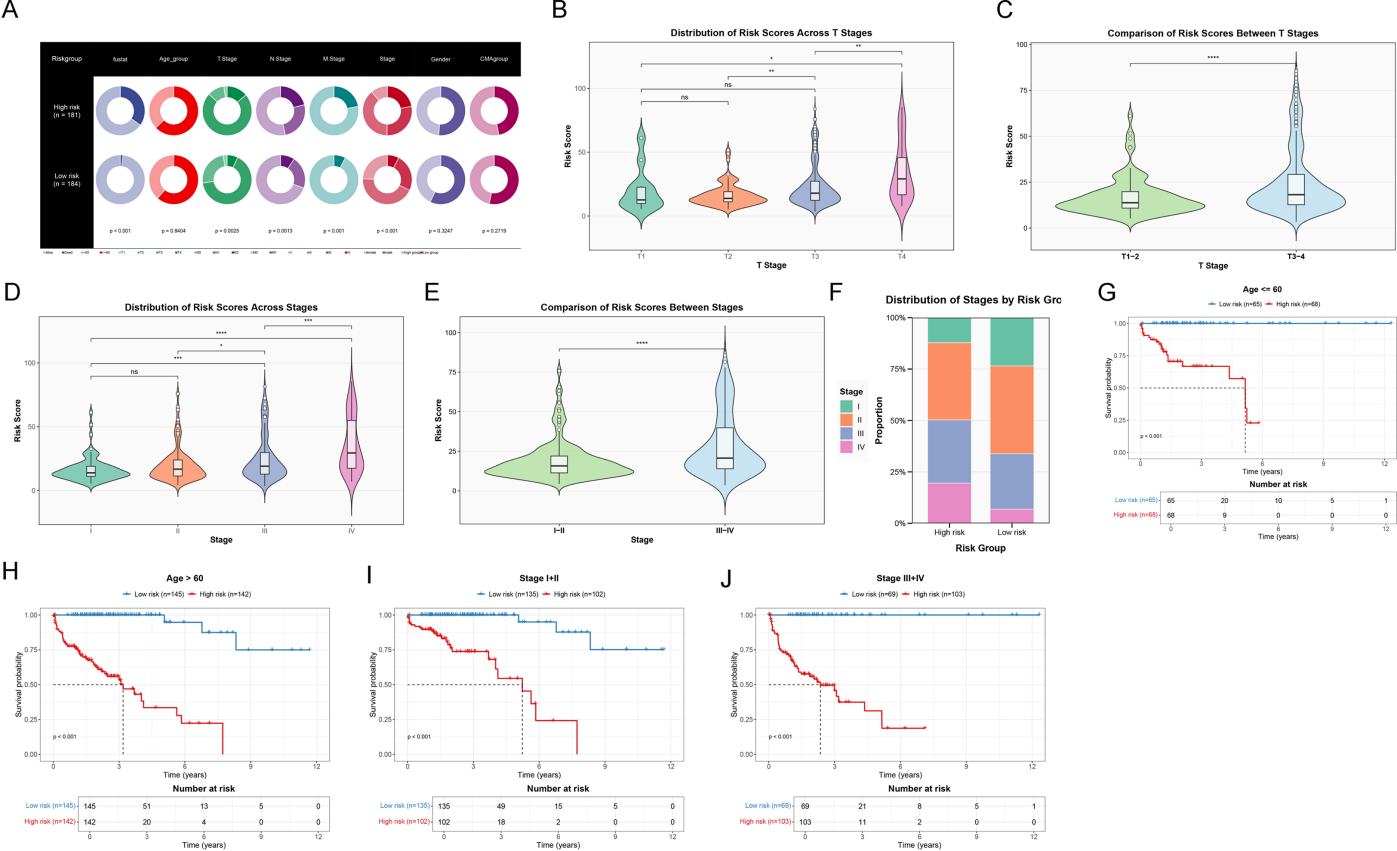
Clinical relevance and stratified survival analysis of the CMA risk model. **(A)** Heatmap illustrating the distribution of clinicopathological features between the high- and low-risk groups. **(B)** Distribution of risk scores across distinct T stages. **(C)** Comparison of risk scores between T1–2 and T3–4 stages. **(D)** Distribution of risk scores across overall clinical stages. **(E)** Comparison of risk scores between Stage I-II and Stage III-IV. **(F)** Proportion of clinical stages within the high- and low-risk groups. **(G)** Kaplan-Meier OS curve for patients aged ≤ 60 years. **(H)** Kaplan-Meier OS curve for patients aged > 60 years. **(I)** Kaplan-Meier OS curve for patients in Stage I-II. **(J)** Kaplan-Meier OS curve for patients in Stage III-IV.

To comprehensively evaluate the applicability of the transcriptome-based CMA prognostic risk model in a real clinical context, we systematically analyzed the associations between the Risk Score and clinicopathological characteristics of patients with COAD. The clinical correlation heatmap showed that patients in the high-risk and low-risk groups displayed markedly heterogeneous distributions in survival status, T stage, N stage, M stage, and overall clinical stage (**Figure 6A**). Specifically, the Risk Score increased significantly with increasing depth of tumor invasion, and patients with T3–4 disease had significantly higher scores than those with T1–2 disease (**Figures 6B, C**). A similar trend was observed across overall clinical stage, with patients in advanced stages (Stage III-IV) showing significantly higher Risk Scores than those in early stages (Stage I-II) (**Figures 6D, E**). In addition, the stage distribution plot further confirmed that the proportion of advanced COAD cases was markedly increased in the high-risk group (**Figure 6F**). These findings indicate that the CMA-related risk model is closely associated with malignant progression and adverse clinical phenotypes in COAD.

To determine whether this model could provide prognostic value independent of conventional clinicopathological features, we next performed stratified Kaplan-Meier survival analyses in different clinical subgroups. The results showed that, regardless of whether patients were younger (≤ 60 years) or older (> 60 years) (**Figures 6G, H**), or whether they were at an early stage (Stage I-II) or an advanced stage (Stage III-IV) (**Figures 6I, J**), OS was consistently and significantly shorter in the high-risk group than in the low-risk group (all subgroup *P* < 0.001). These results strongly support that the CMA prognostic model retains stable and powerful predictive performance independent of patient age and disease stage. However, although this transcriptome-based CMA model exhibited excellent prognostic performance, its strong dependence on large-scale bulk sequencing or single-cell sequencing data substantially limits its broad implementation in routine clinical practice. These findings suggest that CMA-related biological effects may not be fully captured by a global score at the bulk level, but may instead depend more heavily on changes in specific cell subpopulations and microenvironmental composition.

### 3.6 Construction and external validation of the immune risk score

Given that transcriptome-based risk models may be difficult to implement directly in routine clinical practice, and that tumor immune microenvironment features may provide complementary prognostic information, we further constructed an integrated prognostic score based on microenvironmental cell infiltration characteristics and clinicopathological variables.

**Figure 7:**
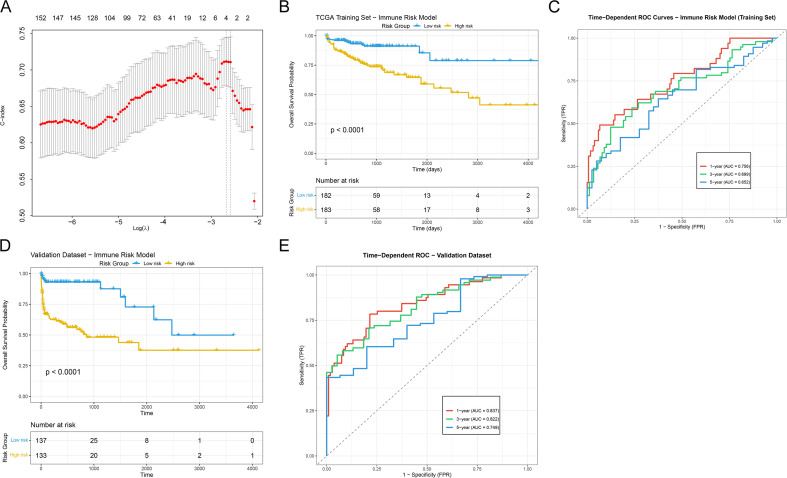
Construction and evaluation of the immune risk score and its cDC-related component. **(A)** LASSO regression analysis for variable selection in the TCGA cohort. **(B)** Kaplan-Meier OS curve of the Immune Risk Score in the TCGA cohort. **(C)** Time-dependent ROC curves of the Immune Risk Score in the TCGA cohort. **(D)** Kaplan-Meier OS curve of the transcriptome-inferred cDC-related component in the single-center cohort from Liaoning Central Hospital. **(E)** Time-dependent ROC curves of the transcriptome-inferred cDC-related component in the single-center cohort from Liaoning Central Hospital.

First, the xCell algorithm was applied to deconvolute the TCGA cohort samples, and the resulting immune and stromal cell infiltration features, together with clinical variables, were incorporated into LASSO Cox regression analysis. The results showed that T stage, M stage, and conventional dendritic cells (cDCs) were ultimately retained as the core variables for constructing the Immune Risk Score (**Figure 7A**). In the TCGA training cohort, patients were stratified into high-risk and low-risk groups according to the Immune Risk Score. Kaplan-Meier survival analysis showed that patients in the high-risk group had significantly shorter OS than those in the low-risk group (*P* < 0.0001) (**Figure 7B**). Time-dependent ROC curve analysis showed that the model achieved AUC values of 0.756, 0.699, and 0.652 for predicting 1-year, 3-year, and 5-year OS, respectively, in the TCGA cohort (**Figure 7C**).

We then performed a single-center evaluation using transcriptomic and clinical data from Liaoning Central Hospital. The available baseline characteristics of this cohort are summarized in **Table 1**. Because complete standardized TNM information was unavailable in this cohort, the full three-variable Immune Risk Score could not be directly validated. Instead, we evaluated the prognostic relevance of the transcriptome-inferred cDC component, which represented the immune microenvironmental variable retained in the TCGA-derived integrated score. Patients stratified by the cDC-related component showed significantly different OS in the single-center cohort, with shorter OS observed in the high-risk group than in the low-risk group (P < 0.0001, **Figure 7D**). Time-dependent ROC curves further supported its predictive performance in this single-center transcriptomic cohort (**Figure 7E**). Taken together, these results suggest that the cDC-related microenvironmental component retained prognostic relevance in the local cohort, although the full three-variable Immune Risk Score requires further validation in cohorts with complete standardized TNM information.

**Table 1:** Baseline characteristics of the single-center cohort stratified by risk group.

Characteristics	Overall (n = 270)	Low risk (n = 137)	High risk (n = 133)	P value
Age, years	68.00 [63.00, 75.00]	68.00 [61.00, 75.00]	69.00 [63.00, 75.00]	0.204
Sex				0.005
Male	179 (66.3)	102 (74.5)	77 (57.9)	
Female	91 (33.7)	35 (25.5)	56 (42.1)	
Survival status				<0.001
Alive	200 (74.1)	124 (90.5)	76 (57.1)	
Dead	70 (25.9)	13 (9.5)	57 (42.9)	
Follow-up time	111.87 [39.83, 719.50]	146.00 [50.10, 730.00]	106.94 [31.00, 662.00]	0.105
Risk score	-3.83 [-4.28, -3.37]	-4.26 [-4.72, -4.01]	-3.36 [-3.57, -2.84]	<0.001

Continuous variables are presented as median [interquartile range], and categorical variables are presented as n (%). P values were calculated using the Wilcoxon rank-sum test for continuous variables and Fisher’s exact test for categorical variables. Survival status was coded as 0 for alive and 1 for dead.

### 3.7 Independence assessment and biological feature comparison of the risk score and the immune risk score

**Figure 8:**
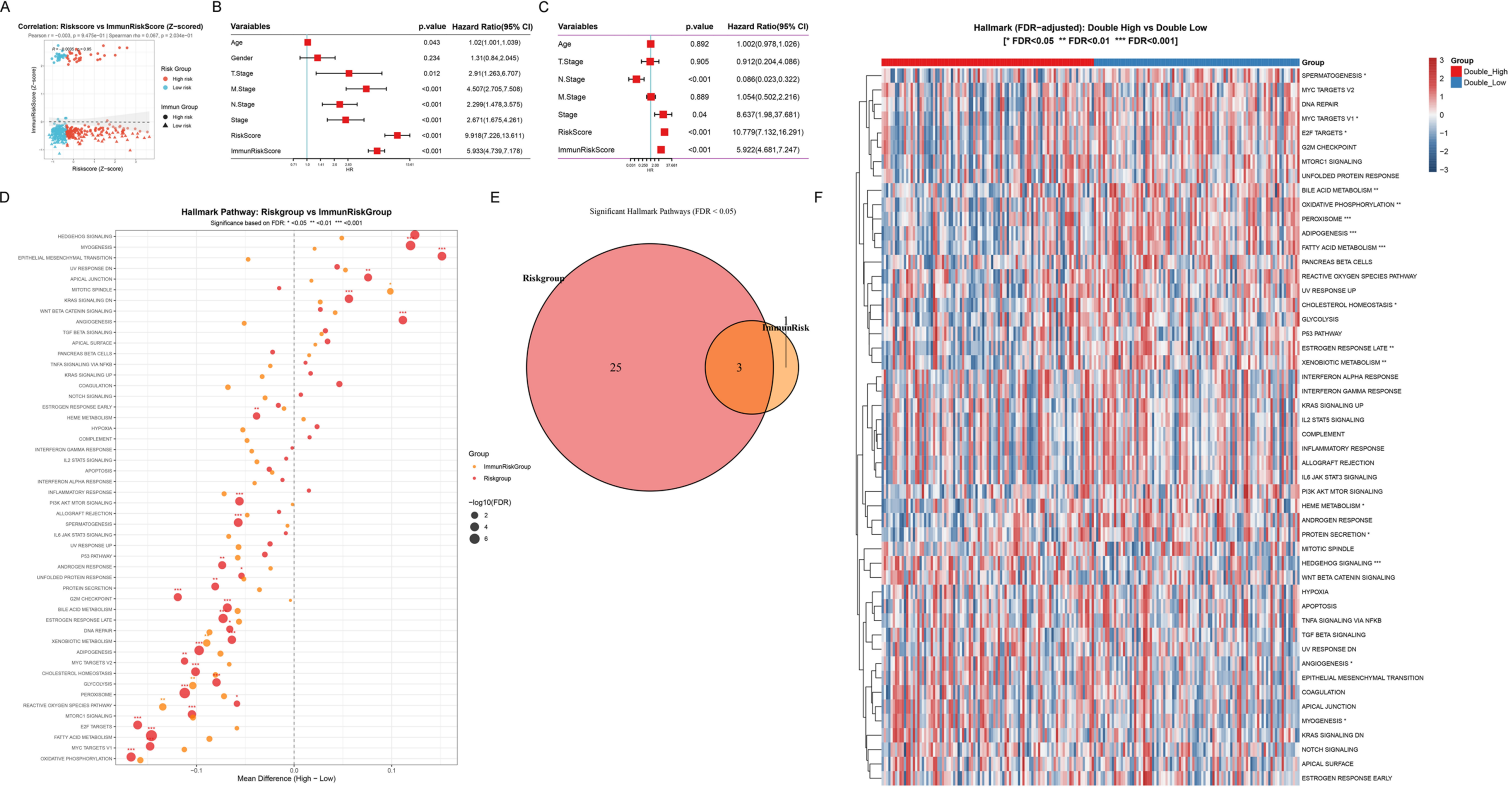
Prognostic assessment and biological pathway features of the risk score and immune risk score. **(A)** Correlation analysis between the genomic Risk Score and the Immune Risk Score. **(B)** Univariate Cox regression analysis of the two risk scores alongside routine clinicopathological features. **(C)** Multivariate Cox regression analysis showing the independent prognostic value of the transcriptome-based Risk Score and the integrated prognostic contribution of the Immune Risk Score. **(D)** Bubble plot comparing the significantly enriched Hallmark pathways between the two risk groups. **(E)** Venn diagram illustrating the shared and uniquely enriched Hallmark pathways (FDR < 0.05). **(F)** Heatmap showing the differences in pathway activation between the “double-high” and “double-low” risk subgroups.

We first performed correlation analysis between the Risk Score and the Immune Risk Score. The results showed no significant linear correlation between the two scores (Pearson *r* = -0.003, *P* = 0.95) (**Figure 8A**), indicating that these two models captured features from different dimensions and could serve as mutually independent prognostic parameters. In univariate Cox regression analysis, both the Risk Score (HR = 9.918, *P* < 0.001) and the Immune Risk Score (HR = 5.933, *P* < 0.001) were significantly associated with OS (**Figure 8B**). Subsequent multivariate Cox regression analysis confirmed the independent prognostic value of the transcriptome-based Risk Score, whereas the Immune Risk Score provided an integrated prognostic assessment by combining T stage, M stage, and cDC-related microenvironmental information(Risk Score: HR = 10.779, *P* < 0.001; Immune Risk Score: HR = 5.922, *P* < 0.001), and that the prognostic weights of both scores in the multivariate model were greater than that of the conventional overall clinical stage (Stage) (**Figure 8C**).

At the biological level, we analyzed and compared Hallmark pathway enrichment patterns between the high-risk and low-risk groups defined by the two scoring systems (**Figure 8D**). The Venn diagram showed that, among significantly enriched pathways (FDR < 0.05), the two models shared three biological pathways, namely XENOBIOTIC METABOLISM, GLYCOLYSIS, and REACTIVE OXYGEN SPECIES PATHWAY. Meanwhile, the Risk Score and the Immune Risk Score showed 25 and 1 uniquely enriched pathways, respectively (**Figure 8E**). Finally, patients were further stratified into double-high (high Risk Score and high Immune Risk Score) and double-low subgroups. Comparative analysis showed that these two extreme subgroups differed significantly in the activity of multiple Hallmark pathways, including Hedgehog signaling and Adipogenesis (**Figure 8F**).

### 3.8 Comparison of immune microenvironmental features between the prognostic models and selection of the key validation gene

**Figure 9:**
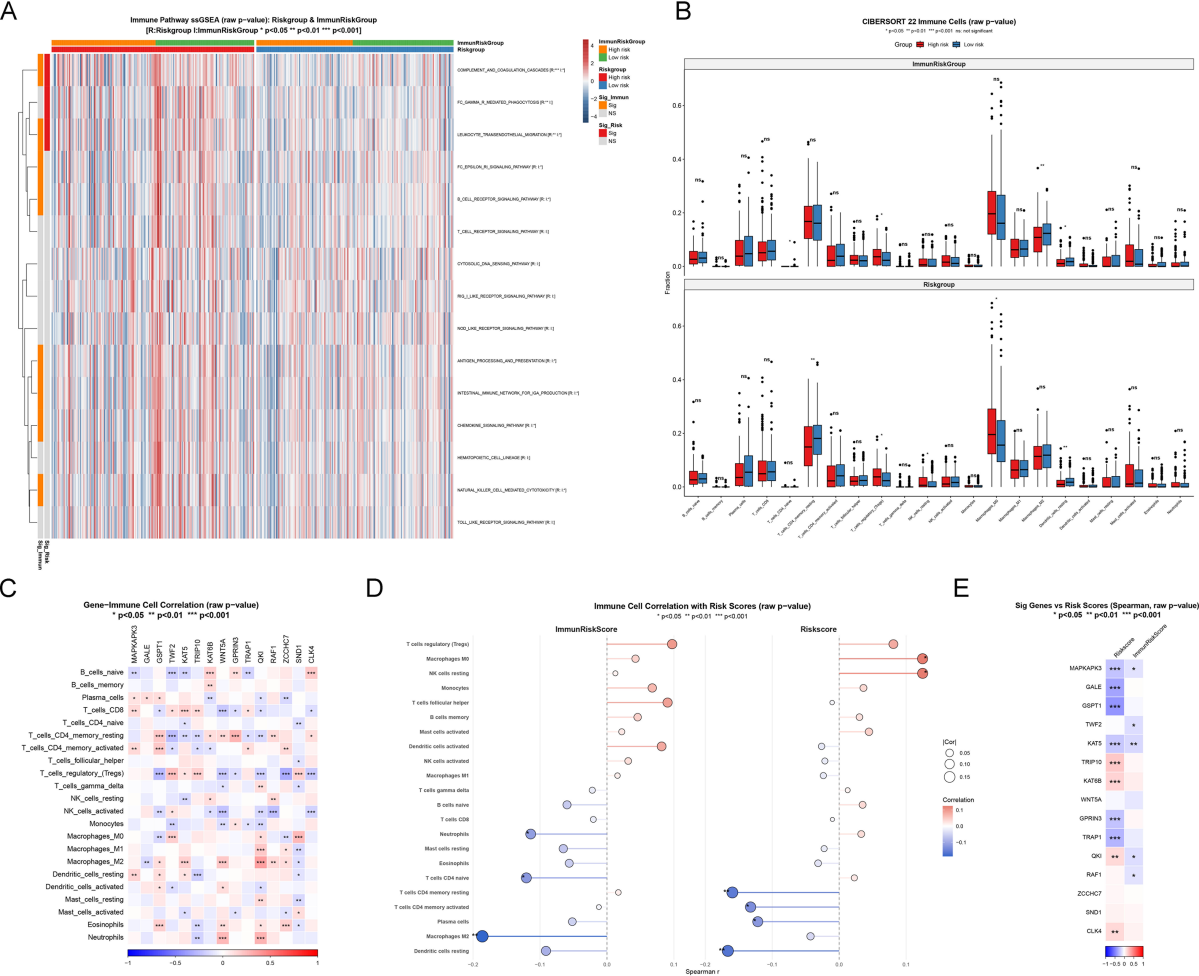
Immune microenvironment landscape and identification of key signature genes. **(A)** Heatmap of ssGSEA scores for immune-related pathways between high- and low-risk groups. **(B)** CIBERSORT analysis comparing the infiltration levels of 22 immune cell types. **(C)** Correlation matrix between the 15 signature genes and specific immune cell subsets. **(D)** Lollipop chart illustrating the correlation between immune cell abundance and the two risk scores. **(E)** Correlation analysis between the 15 signature genes and the two risk scores. *P < 0.05, **P < 0.01, ***P < 0.001.

First, we evaluated the enrichment of immune-related pathways in different risk groups using ssGSEA. The heatmap showed that although the two scoring models differed to some extent in their characterization of the microenvironment, two key immune pathways, Complement and coagulation cascades and LEUKOCYTE TRANSENDOTHELIAL MIGRATION, showed significant enrichment differences between the high-risk and low-risk groups in both the Risk Score and Immune Risk Score models (**Figure 9A**). We then quantified the relative infiltration abundance of 22 immune cell subsets in the tumor microenvironment using the CIBERSORT algorithm. Differential analysis further confirmed the convergence of these shared significant features. Among all detected immune cell subsets, only T cells regulatory (Tregs) and Dendritic cells resting showed significant differences in infiltration abundance across the risk groups defined by both the Risk Score and the Immune Risk Score (**Figure 9B**).

To explore the direct molecular associations between the 15 feature genes used for model construction and local immune infiltration, we performed gene-immune cell correlation analysis (**Figure 9C**). The results showed that among these 15 genes, MAPKAPK3 exhibited broader and more stable significant correlations with multiple immune cell subsets, including B cells naive, T cells CD8, and Dendritic cells resting. Notably, Dendritic cells resting also showed consistent cross-model differences in the previous analysis, further suggesting that the genes highly correlated with this cell type may play a potentially important role in local microenvironmental regulation. At the level of global risk scores, we further assessed the specific associations between the two risk scores and the infiltration abundance of different immune cell subsets (**Figure 9D**). Correlation analysis showed that the Risk Score was significantly positively correlated with Macrophages M0 and NK cells resting, and significantly negatively correlated with T cells CD4 memory resting, Dendritic cells resting, T cells CD4 memory activated, and Plasma cells. In contrast, the Immune Risk Score was mainly significantly negatively correlated with Macrophages M2, Neutrophils, and T cells CD4 naive.

Finally, we analyzed the associations between the 15 feature genes and the two risk scores themselves (**Figure 9E**). The correlation results clearly showed that MAPKAPK3 was the most prominent gene, exhibiting highly significant correlations with both independent risk models. Considering its strong consistency across the dual prognostic models and its close association with key microenvironmental cells, such as Dendritic cells resting, we ultimately selected MAPKAPK3 as the key target for subsequent *in vitro* validation in cell lines.

### 3.9 *In vitro* functional validation of the core feature gene MAPKAPK3 in COAD cells

**Figure 10:**
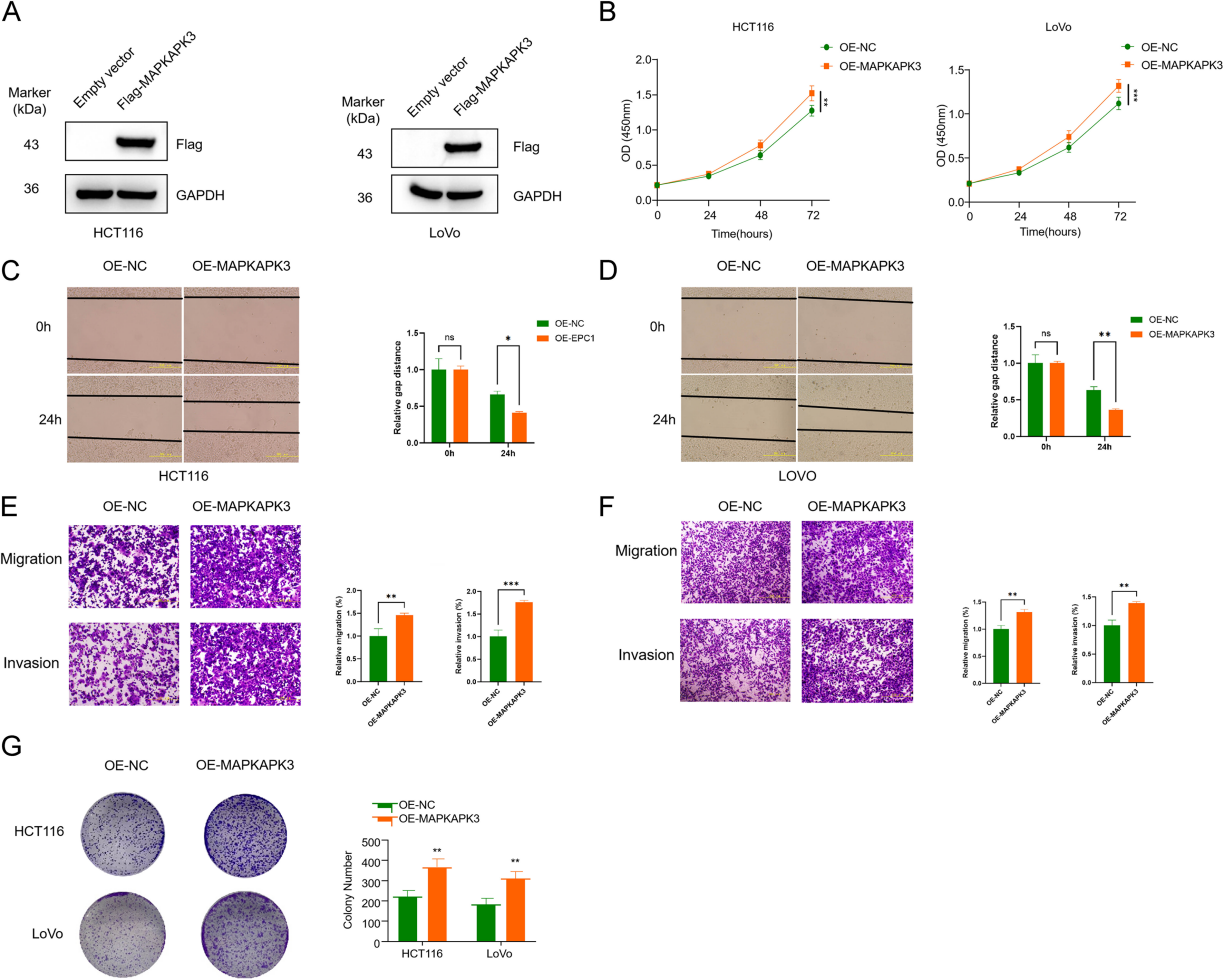
MAPKAPK3 promotes malignant phenotypes in COAD cells. **(A)** Western blot analysis confirmed successful overexpression of MAPKAPK3 in HCT116 and LoVo cells. GAPDH was used as a loading control. **(B)** CCK-8 assays showed that MAPKAPK3 overexpression significantly enhanced cell proliferation in HCT116 and LoVo cells. **(C)** Wound-healing assay demonstrated that MAPKAPK3 overexpression promoted migration of HCT116 cells. **(D)** Wound-healing assay showed that MAPKAPK3 overexpression enhanced migration ability in LoVo cells. **(E)** Transwell assays indicated that MAPKAPK3 overexpression significantly increased migration and invasion in HCT116 cells. **(F)** Transwell assays showed that MAPKAPK3 overexpression enhanced migration and invasion in LoVo cells. **(G)** Colony formation assays revealed that MAPKAPK3 overexpression significantly promoted clonogenic ability in both HCT116 and LoVo cells. *P < 0.05, **P < 0.01, ***P < 0.001, ns not significant.

Among the CMA-related prognostic candidate genes, MAPKAPK3 ranked as the top feature according to RSF variable importance and showed consistent associations with the dual-risk models and immune infiltration features. In addition, relatively few studies have investigated the functional role of MAPKAPK3 in COAD. Therefore, MAPKAPK3 was selected for subsequent *in vitro* validation. MAPKAPK3 overexpression models were established in HCT116 and LoVo cell lines. Western blot results showed that, compared with the empty vector group, MAPKAPK3 protein expression was markedly increased in the Flag-MAPKAPK3 group, and stable overexpression was observed in both cell lines (**Figure 10A**).

Cell proliferation assays showed that MAPKAPK3 overexpression significantly enhanced the proliferative capacity of both HCT116 and LoVo cells. CCK-8 results demonstrated that, in HCT116 cells, the OD450 value at 72 h was significantly higher in the OE-MAPKAPK3 group than in the control group (*P* < 0.01). In LoVo cells, the OD450 value at 72 h was also significantly higher in the OE-MAPKAPK3 group than in the control group (*P* < 0.001) (**Figure 10B**).

Wound-healing assays were further performed to evaluate the effect of MAPKAPK3 on cell migration. In HCT116 cells, the relative wound distance at 24 h was significantly lower in the OE-MAPKAPK3 group than in the OE-NC group (*P* < 0.05). In LoVo cells, wound closure at 24 h was significantly accelerated in the OE-MAPKAPK3 group (*P* < 0.01) (**Figures 10C, D**).

Transwell assay results showed that MAPKAPK3 overexpression significantly enhanced both migration and invasion in HCT116 cells, with both differences reaching statistical significance (*P* < 0.01 and *P* < 0.001, respectively) (**Figure 10E**). A similar trend was observed in LoVo cells, in which the numbers of migratory and invasive cells were both significantly higher in the OE-MAPKAPK3 group than in the control group (both *P* < 0.01) (**Figure 10F**).

Colony formation assays showed that, compared with the control group, the OE-MAPKAPK3 group formed significantly more colonies in both HCT116 and LoVo cells, and the differences were statistically significant in both cell lines (both *P* < 0.01) (**Figure 10G**).

Taken together, these results indicate that MAPKAPK3 exerts a tumor-promoting role in COAD cells and can significantly enhance cell proliferation, migration, and invasion, although its direct regulatory relationship with CMA activity remains to be further validated.

### 3.10 Evaluation of the two prognostic models in predicting drug sensitivity

**Figure 11:**
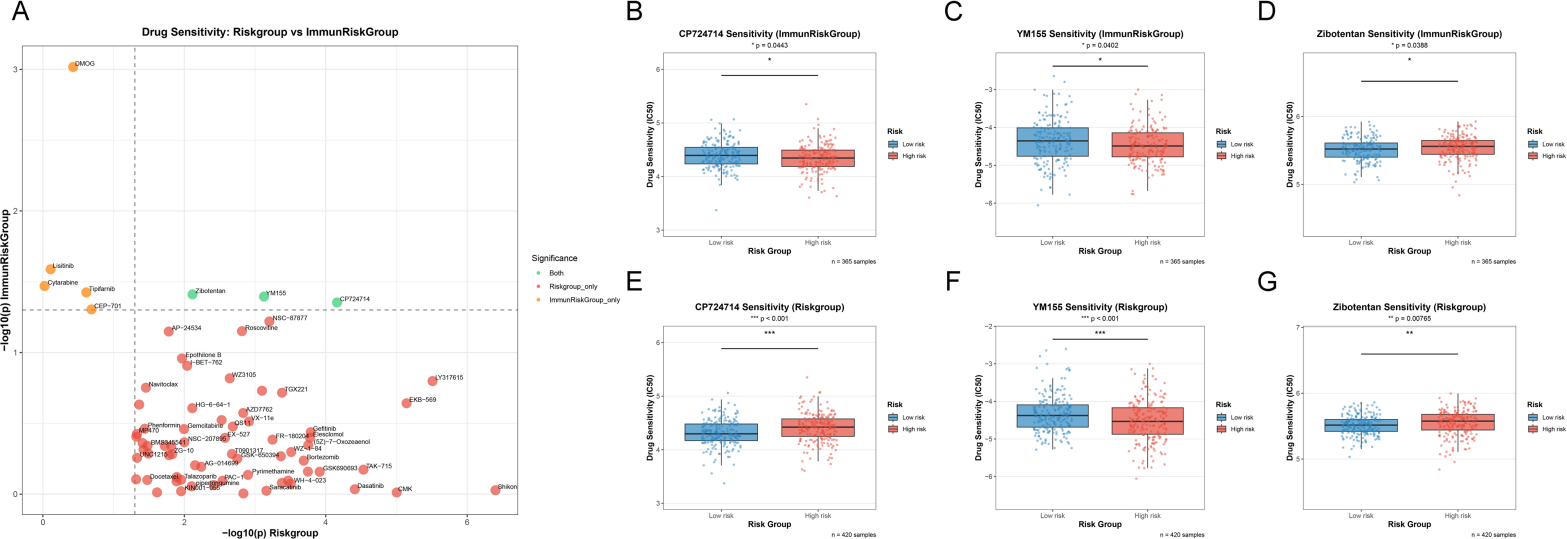
Evaluation of drug sensitivity based on the two prognostic models. **(A)** Scatter plot comparing the statistical significance of drug sensitivity differences between risk groups across both models. **(B)** Estimated IC50 values of CP724714 between the high and low Immune Risk Score groups. **(C)** Estimated IC50 values of YM155 between the high and low Immune Risk Score groups. **(D)** Estimated IC50 values of Zibotentan between the high and low Immune Risk Score groups. **(E)** Estimated IC50 values of CP724714 between the high and low Risk Score groups. **(F)** Estimated IC50 values of YM155 between the high and low Risk Score groups. **(G)** Estimated IC50 values of Zibotentan between the high and low Risk Score groups. *P < 0.05, **P < 0.01, ***P < 0.001.

To explore whether the two prognostic models were associated with potential therapeutic vulnerabilities, we performed an exploratory drug sensitivity prediction analysis using all available drugs in the CGP2016 database through the pRRophetic framework. First, scatter plots were used to systematically compare the statistical significance of differences in predicted IC50 values for multiple drugs between the high-risk and low-risk groups defined by the Risk Score and the Immune Risk Score (**Figure 11A**). The results showed that some targeted agents exhibited significant differences in predicted sensitivity in both independent risk assessment systems.

We then focused on three representative drugs, Zibotentan, YM155, and CP724714, for detailed quantitative comparison. Specifically, for Zibotentan, the predicted IC50 values were significantly higher in the high-risk group than in the low-risk group in both models (Immune Risk Score: *P* = 0.0388, **Figure 11D**; Risk Score: *P* = 0.00765, **Figure 11G**), indicating that the low-risk population might be more sensitive to this drug. In contrast, YM155 showed the opposite trend, with significantly higher predicted IC50 values in the low-risk group than in the high-risk group in both models (Immune Risk Score: *P* = 0.0402, **Figure 11C**; Risk Score: *P* < 0.001, **Figure 11F**), suggesting that the high-risk population might be more sensitive to YM155. Notably, the predicted sensitivity to CP724714 showed a certain degree of heterogeneity between the two systems. In the Immune Risk Score model, the predicted IC50 value was significantly higher in the low-risk group than in the high-risk group (*P* = 0.0443, **Figure 11B**), whereas in the Risk Score model, the predicted IC50 value was significantly lower in the low-risk group than in the high-risk group (*P* < 0.001, **Figure 11E**). These results suggest that patients with different risk stratifications may exhibit distinct predicted drug sensitivity patterns, although these findings remain computational and hypothesis-generating.

## 4 Discussion

By integrating single-cell transcriptomics, bulk transcriptomics, and ensemble machine learning, this study systematically revealed CMA-related heterogeneity in COAD and established a dual prognostic framework evaluated through cross-cohort assessment and single-center transcriptomic analysis. Unlike most previous studies that focused on a single gene, a single cohort, or a single algorithm, the major strength of this work lies in the integration of cell-subpopulation heterogeneity at single-cell resolution, robust feature selection at the bulk level, cross-cohort machine learning modeling, immune microenvironment characterization, and *in vitro* functional validation into a more comprehensive evidence framework (33–36). More importantly, CMA was not treated as a single averaged molecular event. Instead, our results suggest that its biological and clinical significance may depend more on the remodeling of specific cell subpopulations, particularly within the myeloid compartment, rather than being fully captured by bulk-level global expression (37).

Liu et al. summarized that CMA contributes to metabolic adaptation, protein homeostasis maintenance, and stress survival in tumors, and is closely associated with tumor progression and immune regulation (38). However, in COAD, most studies on CMA have remained limited to individual molecules or *in vitro* phenotypes, with a lack of systematic analyses addressing its heterogeneity and microenvironmental relevance at single-cell resolution (39, 40). In this study, CMA activity was not uniformly distributed across cell types but was more prominently enriched in myeloid cells, suggesting that the role of CMA in COAD may be more closely linked to regulation of the tumor microenvironment, particularly the functional states of myeloid cells (41, 42). Further myeloid subpopulation analysis showed that CMA-related signals were not uniformly increased across all myeloid cells, but were accompanied by shifts in the composition and state of different subpopulations. These findings suggest that CMA may be more closely involved in the functional remodeling of myeloid cells and the establishment of an immunosuppressive microenvironment than in tumor cell-intrinsic metabolic programs alone (43).

Our findings further suggest that risk stratification in COAD cannot be adequately explained from a single dimension, but instead requires simultaneous consideration of tumor-intrinsic molecular programs and immune microenvironmental status (44, 45). Unlike traditional single-score models, this study established two independent yet complementary prognostic systems, namely the Risk Score and the Immune Risk Score. The Risk Score was derived mainly from molecular features selected from candidate genes through large-scale ensemble machine learning and was therefore more reflective of tumor-intrinsic transcriptomic abnormalities. In contrast, the Immune Risk Score was constructed from immune microenvironmental components and clinical variables, and thus primarily captured immune microenvironmental and clinical progression-related features. Although the two scores showed weak correlation, they reflected different layers of prognostic information. The transcriptome-based Risk Score captured molecular risk features, whereas the Immune Risk Score represented a composite prognostic model integrating T stage, M stage, and cDC-related microenvironmental information. Notably, the Immune Risk Score was ultimately composed of T stage, M stage, and cDCs. Therefore, this score should be interpreted as a clinicopathological-immune integrated prognostic score rather than a purely immune-derived marker. Its prognostic performance is partly attributable to the contributions of T and M stages, while cDCs provide additional transcriptome-inferred microenvironmental information. Compared with T stage and M stage, which mainly reflect the extent of the primary tumor and the presence of distant metastasis, respectively, the inclusion of cDCs suggests that immune microenvironmental features themselves can provide additional prognostic information in COAD (46). Dendritic cells are critical antigen-presenting cells linking innate and adaptive immunity, and their abundance and functional status can directly affect the initiation of antitumor immune responses (47, 48). In this study, a higher cDCs level was associated with a lower risk tendency, suggesting that it may reflect a microenvironmental state more permissive to antitumor immune activation. This finding further supports the view that prognostic evaluation in COAD should not rely solely on tumor-intrinsic molecular abnormalities, but should also consider the microenvironmental states represented by key immune cell subsets.

MAPKAPK3 was ultimately selected as the key validation gene primarily because it ranked as the top feature in the RSF variable-importance analysis and showed consistent associations with the dual-risk models and immune infiltration features. This selection strategy was intended to prioritize a prognostically informative and biologically relevant candidate for experimental validation. Previous studies have suggested that MAPKAPK3, as a downstream kinase of the p38 mitogen-activated protein kinase (p38 MAPK) pathway, is a downstream kinase of the p38 mitogen-activated protein kinase pathway and has been implicated in inflammatory signaling, stress responses, and other cancer-related biological processes (49, 50). Our study further demonstrated that MAPKAPK3 overexpression significantly promoted the proliferation, migration, and invasion of COAD cells, supporting its tumor-promoting role in COAD progression. Combined with the immune-related findings of this study, MAPKAPK3 may not only affect tumor cell proliferation and migration directly, but may also contribute to adverse disease progression by influencing tumor-immune interactions within the microenvironment (51). However, MAPKAPK3 was selected based on machine-learning importance, dual-score correlations, and immune-infiltration associations. Therefore, its relationship with CMA-related activity should be interpreted as transcriptomic and correlative rather than mechanistically causal. Direct experimental evidence using CMA reporter systems, LAMP2A-based assays, lysosomal fractionation, or functional CMA flux assays will be required to determine whether MAPKAPK3 directly regulates CMA activity in colon cancer.

Several limitations should be acknowledged. First, most findings were derived from public datasets. Although multicohort and single-center validations were performed, further confirmation in larger, multicenter, prospective cohorts is still needed, consistent with broader colorectal cancer biomarker research showing that small sample sizes, selection bias, and limited generalizability remain major barriers to clinical translation (52). In addition, the frequency-based single-cell filtering strategy prioritized recurrent CMA-related genes shared by at least two myeloid subpopulations, which may have excluded rare but biologically meaningful lineage-specific signals. Future studies focusing on individual myeloid subpopulations are needed to further clarify these subtype-specific mechanisms. Second, the *in vitro* experiments were limited to MAPKAPK3 overexpression. Although these gain-of-function assays provided preliminary evidence that MAPKAPK3 may promote malignant phenotypes in colon cancer cells, knockdown or rescue experiments and *in vivo* tumor growth or metastasis models were not performed. Therefore, the oncogenic role and mechanistic relevance of MAPKAPK3 should be interpreted cautiously and require further validation. Third, although the Immune Risk Score showed favorable prognostic stratification ability, the biological mechanisms underlying its immune-related parameters remain to be experimentally clarified, particularly the specific role of cDCs in immune regulation in COAD. Finally, the drug sensitivity analysis was computational and hypothesis-generating. The identified agents are not standard-of-care therapies for colon cancer, and biologic agents such as anti-EGFR antibodies are not adequately captured by the CGP2016/pRRophetic framework. Overall, this study provides a relatively comprehensive depiction of CMA-related biological processes in colon cancer from four aspects, namely single-cell heterogeneity, dual-score modeling, single-center validation, and functional experiments, and suggests that the combined application of the Risk Score and the Immune Risk Score may offer a new framework for risk stratification and individualized therapeutic exploration in COAD.

## 5 Conclusion

This study systematically revealed CMA-related heterogeneity in COAD and established a robust dual prognostic evaluation system. The transcriptome-based Risk Score showed independent prognostic value, whereas the Immune Risk Score provided a complementary integrated prognostic model based on clinicopathologic and immune microenvironmental features. MAPKAPK3 was further identified and experimentally validated as a tumor-promoting gene associated with the CMA-related prognostic framework, suggesting that it may play an important role in COAD progression and immune microenvironment remodeling. These findings provide a new basis for risk stratification and individualized therapeutic exploration in COAD.

## Data Availability

The original contributions presented in the study are included in the article/**Supplementary Material**. Further inquiries can be directed to the corresponding author/s.
