# Supplementary figures and images for: Integrative multi-omics analysis identifies a CMA-associated heterogeneity risk score and a cDCs-based immune score for robust prognostic stratification in colon cancer with single-center and experimental validation
Source: Front Immunol. 2026 Jun 3;17:1838405. doi: 10.3389/fimmu.2026.1838405 (PMC13272346; doi:10.3389/fimmu.2026.1838405)

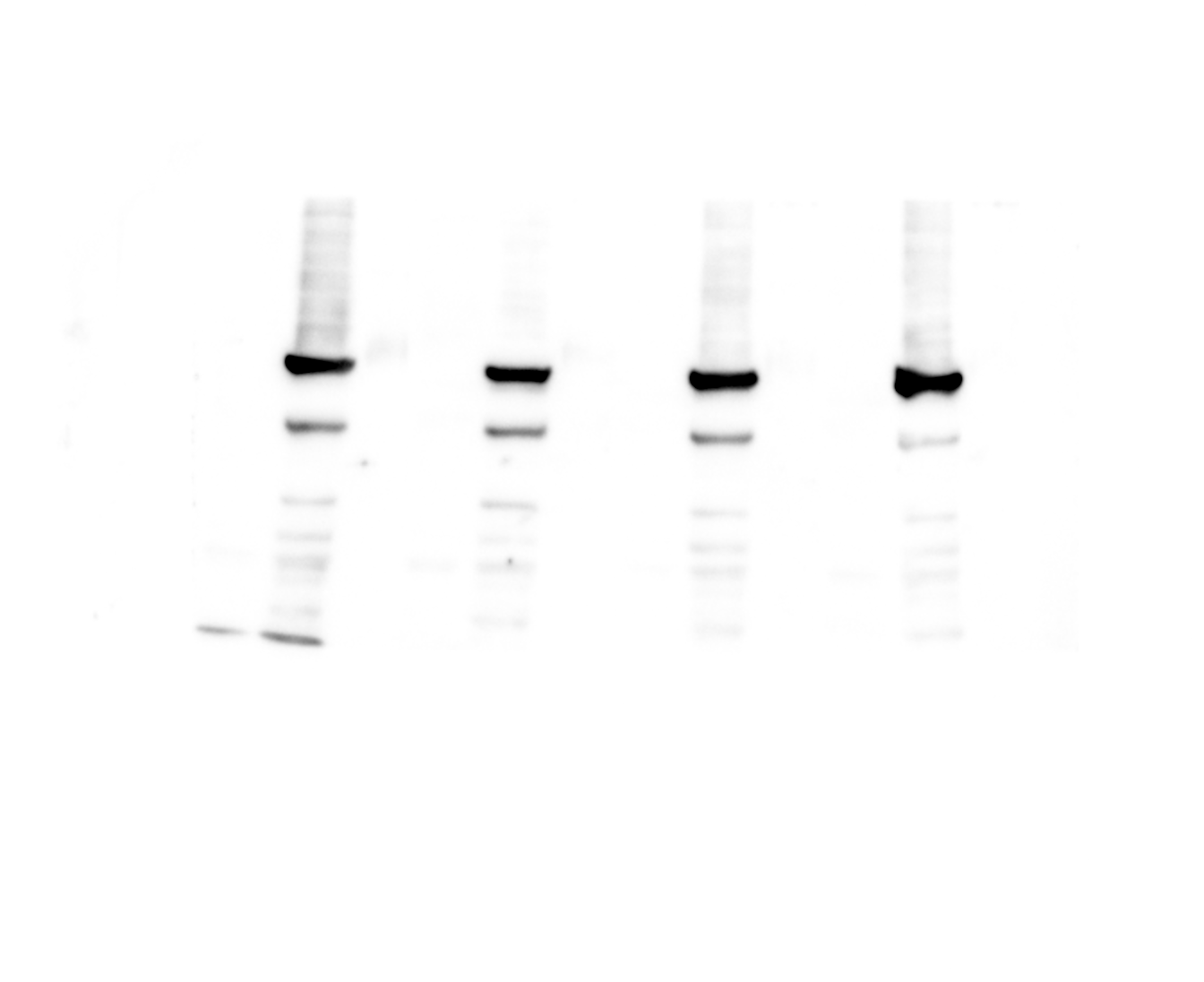

Supplement: Supplementary file 1 [file Image1.tif]

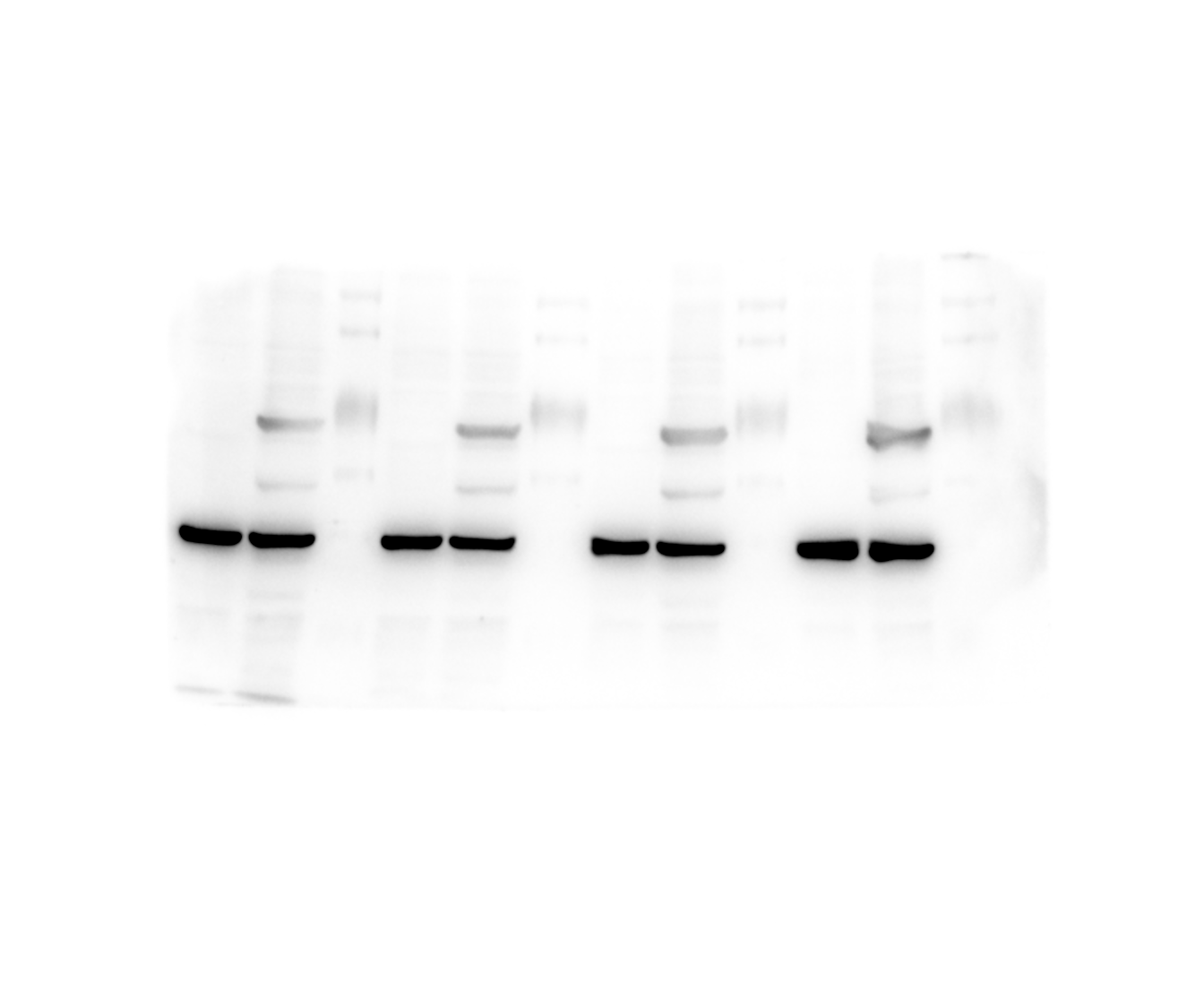

Supplement: Supplementary file 2 [file Image2.tif]

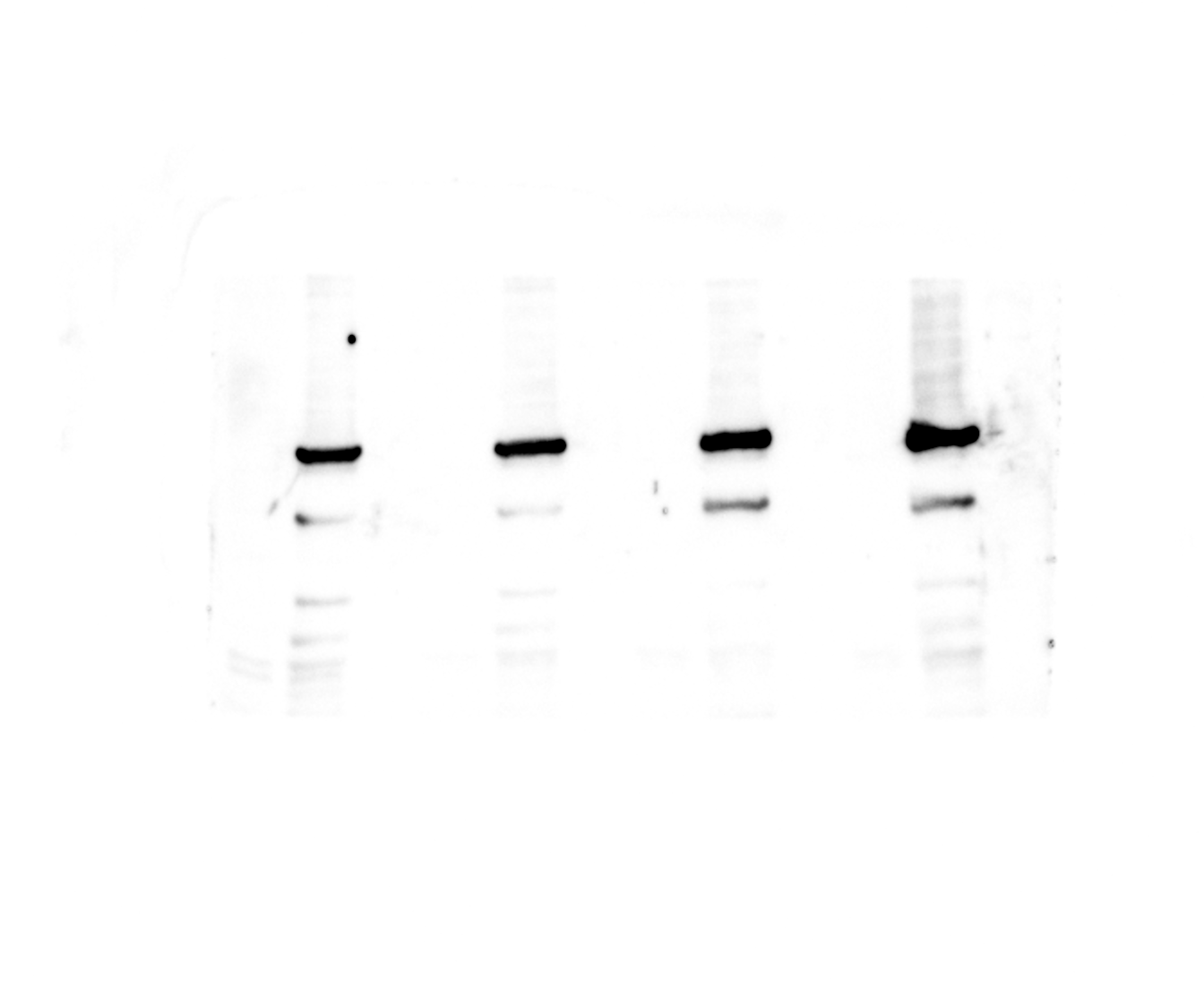

Supplement: Supplementary file 3 [file Image3.tif]

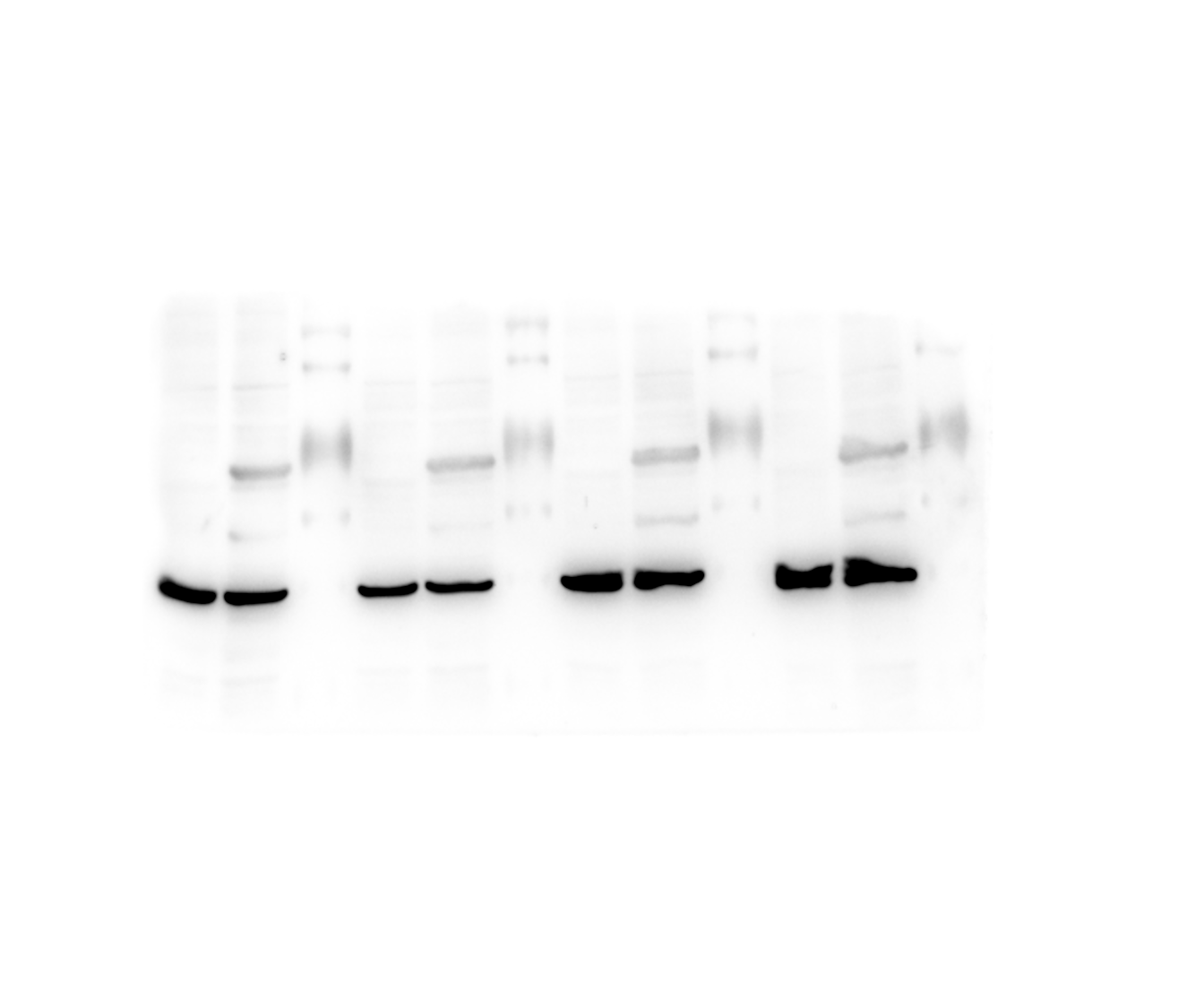

Supplement: Supplementary file 4 [file Image4.tif]
